# Promoting endogenous articular cartilage regeneration using extracellular matrix scaffolds

**DOI:** 10.1016/j.mtbio.2022.100343

**Published:** 2022-07-05

**Authors:** David C. Browe, Ross Burdis, Pedro J. Díaz-Payno, Fiona E. Freeman, Jessica M. Nulty, Conor T. Buckley, Pieter A.J. Brama, Daniel J. Kelly

**Affiliations:** a Trinity Centre for Biomedical Engineering, Trinity Biomedical Sciences Institute, Trinity College Dublin, Ireland; b Department of Mechanical, Manufacturing and Biomedical Engineering, School of Engineering, Trinity College Dublin, Ireland; c Advanced Materials and Bioengineering Research Centre (AMBER), Royal College of Surgeons in Ireland and Trinity College Dublin, Ireland; d Department of Anatomy and Regenerative Medicine, Royal College of Surgeons in Ireland, Dublin, Ireland; e Section of Veterinary Clinical Sciences, School of Veterinary Medicine, University College Dublin, Ireland; f Institute for Medical Engineering and Science Massachusetts Institute of Technology Cambridge, MA, 02142, USA; g Department of Medicine Division of Engineering in Medicine Brigham and Women’s Hospital Harvard Medical School Boston, MA, 02115, USA

**Keywords:** Cartilage repair, ECM scaffold, Large animal model, Microfracture, Fixation device

## Abstract

Articular cartilage defects fail to heal spontaneously, typically progressing to osteoarthritis. Bone marrow stimulation techniques such as microfracture (MFX) are the current surgical standard of care; however MFX typically produces an inferior fibro-cartilaginous tissue which provides only temporary symptomatic relief. Here we implanted solubilised articular cartilage extracellular matrix (ECM) derived scaffolds into critically sized chondral defects in goats, securely anchoring these implants to the joint surface using a 3D-printed fixation device that overcame the need for sutures or glues. *In vitro* these ECM scaffolds were found to be inherently chondro-inductive, while *in vivo* they promoted superior articular cartilage regeneration compared to microfracture. In an attempt to further improve the quality of repair, we loaded these scaffolds with a known chemotactic factor, transforming growth factor (TGF)-β3. *In vivo* such TGF-β3 loaded scaffolds promoted superior articular cartilage regeneration. This study demonstrates that ECM derived biomaterials, either alone and particularly when combined with exogenous growth factors, can successfully treat articular cartilage defects in a clinically relevant large animal model.

## Graphical abstract

**Figure:**
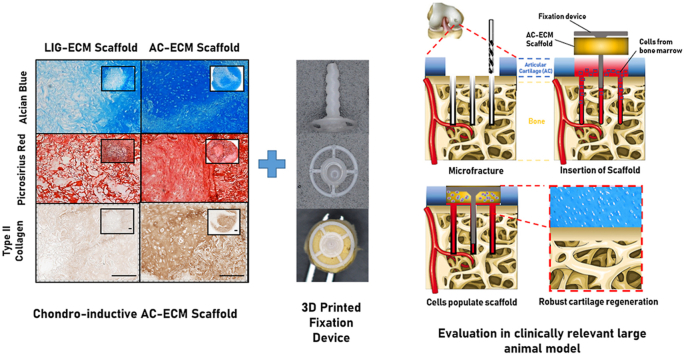

## 1 Introduction

The successful, long-term treatment of articular cartilage injuries remains a major challenge in the field of orthopaedic medicine [1,2]. Articular cartilage has a limited intrinsic regenerative capability post-trauma and as such surgical intervention is necessary to induce repair before focal cartilage lesions can progress to further degeneration and osteoarthritis (OA) [3,4]. OA is one of the leading causes of disability worldwide and represents a massive socioeconomic burden on global populations [5,6]. The prevalence of OA is on the increase and will continue to grow as the mean age of the world population increases over the coming decades [5,7]. The successful treatment of focal cartilage defects is therefore critical to reduce the prevalence of OA [8]. Despite over thirty years of advances in cartilage repair procedures, microfracture (MFX) remains the first line treatment choice for many orthopaedic surgeons due to its low price-point and short surgical time [9,10]. However, the repair tissue generated as a result of MFX generally consists of biomechanically inferior fibrocartilage [9], which is associated with high rates of revision surgery. In addition, initial MFX treatment also reduces the chance of success of subsequent cell based therapeutic options such as autologous chondrocyte transplantation (ACI) or matrix assisted autologous chondrocyte transplantation (MACI) [11,12]. MACI is currently the most common scaffold and cell combination therapy in clinical use and has been shown to promote superior quality repair compared to MFX [13,14]. MACI combines the patient's autologous chondrocytes, which are harvested in an initial surgery, expanded *ex vivo*, and combined with a type I/III collagen-based scaffold which is subsequently implanted during a second surgery several weeks later [15,16]. The high cost associated with multiple surgeries and the expansion of autologous chondrocytes under good manufacturing practice (GMP) conditions has resulted in limited uptake by surgeons and healthcare providers, with MACI being withdrawn from some EU markets due to a lack of reimbursement [17]. The use of osteochondral autograft/allograft transplantation is another available treatment option for the repair of chondral defects [1]. This technique involves the transplantation of an osteochondral plug consisting of viable hyaline cartilage on top of bone from either the patient themselves or a cadaveric donor. Such treatments allow patients to return to activity faster than other treatments but are not without disadvantages such as donor site morbidity (autograft transplantation) [1] or lack of available donors, high cost and risk of disease transmission (allograft transplantation) [18]. Therefore, there is an unmet clinical need for the development of a single-stage, off-the-shelf, cartilage repair therapy that is cost effective, has a short duration of surgery and results in consistent, hyaline-like cartilage repair. A viable option may be to augment or enhance microfracture with the use of scaffolds. This is due to the cost effectiveness of the procedure, the availability of microfracture and the similarity in outcomes that could be obtained when compared to cell-based procedures [[19], [20], [21]].

Extracellular matrix (ECM) derived scaffolds have been shown to promote tissue repair by providing both structural and functional cues to cells [22,23]. Currently the majority of cartilage repair therapies in clinical use are based on biomaterials rich in type I collagen [24]. However, biomaterials derived from type I collagen are not believed to be inherently chondro-inductive, supporting inferior chondrogenesis to type II collagen [25,26], the dominant collagen type within hyaline cartilage. ECM scaffolds fabricated from articular cartilage are naturally rich in type II collagen and such biomaterials have been shown to support robust chondrogenesis both *in vitro* and *in vivo* [[27], [28], [29], [30], [31]]. While the type of collagen present in such ECM scaffolds is a likely driver of chondrogenic differentiation, the ECM itself has been found to contain a milieu of different collagens, proteoglycans, ECM glycoproteins, ECM-regulatory proteins, ECM-affiliated proteins and secreted factors which can play key roles in promoting cartilage repair [32]. In comparison to the vast amount of research performed using type I collagen based scaffolds, relatively little is known about the capacity of cartilage ECM derived biomaterials to promote the regeneration of chondral defects (defects to the articular surface of synovial joints that are not believed to affect the subchondral bone), the most common cartilage injury observed clinically during arthroscopy of the knee joint [33]. The reasons for this are multi-faceted, from the challenge of creating mechanically functional ECM derived scaffolds to the difficulties associated with the fixation of such implants within focal cartilage defects. Scaffold fixation remains a key issue for orthopaedic surgeons due to the shallow nature of the defects themselves and the high biomechanical shear forces encountered in the joint. Glues and sutures are the most common fixation methods employed clinically, however these options are sub optimal, prolong surgery time and are liable to fail [34,35]. Rigid pin fixation of scaffolds and tissue engineered constructs has been performed with varying levels of success [34,36,37].

The overall goal of this study was to develop a cell-fee, single-stage, ‘off-the-shelf’ regenerative implant for articular cartilage repair that did not require ineffective gluing or suturing to stably anchor the device within the synovial joint. To realise this goal, we first developed highly porous and elastic scaffolds from solubilised articular cartilage, demonstrating that such biomaterials support superior chondrogenesis of human bone marrow stromal cells (MSCs) compared to ECM scaffolds derived from solubilised ligament (a type I collagen rich biomaterial)*.*In parallel, we designed and 3D printed a biodegradable fixation device to quickly and securely anchor the scaffold within an articular cartilage defect. We then assessed the capacity of such articular cartilage (AC)-ECM derived scaffolds to augment and improve the outcomes of the microfracture procedure, the current surgical standard of care for cartilage defects, in a clinically relevant caprine model of chondral defect repair. Recognising that the success of such cell-free, biomaterial-based strategies for regenerating damaged tissues relies on the recruitment of endogenous cells into the scaffold post-implantation, we also assessed if functionalization of such implants with a chemotactic growth factor would further improve the regenerative process. We hypothesized that such scaffolds would promote the development of a repair tissue that recapitulated the zonal architecture and composition of normal articular cartilage.

## 2 Materials and methods

### 2.1 Study design

This study was designed to the test the ability of the AC-ECM scaffolds to promote chondrogenic differentiation of human BM-MSCs *in vitro* and having established the chondo-inductivity of these scaffolds to test their ability to promote articular cartilage regeneration in a clinically relevant animal model. All animal experiments were conducted in accordance with the recommendations and guidelines of The Health Products Regulatory Authority, the competent authority in Ireland responsible for the implementation of Directive 2010/63/EU on the protection of animals used for scientific purposes in accordance with the requirements of the Statutory Instrument No. 543 of 2012. All animal experiments were approved by the University College Dublin Animal Research Ethics Committee (Approval number – AREC 12–74) and the Irish Health Products Regulatory Authority (Approval number - AE18982/P032). The *n* for the goats used in this study was based on the predicted variance in the model and was powered to detect 0.05 significance. The implantation groups were randomised across the operated animals. *n* ​= ​9 animals were operated on for each group. Unfortunately, 3 animals from the TGF cohort and 2 animals from the MFX/MFX ​+ ​AC-ECM cohort died prior to the 6-month end-point. These animal deaths were deemed not to be related to the surgical procedure following post-mortem by a veterinarian. Blinded scoring of gross morphology and histology was performed. For this scoring, the samples were randomised using a randomization algorithm and the identification numbers of the animals removed from the samples.

### 2.2 Articular cartilage ECM (AC-ECM) scaffold fabrication

Scaffolds were fabricated as previously described [28]. Briefly, pepsin solubilised porcine articular cartilage (10 ​mg/ml) was cross-linked with glyoxal (5 ​mM), poured into moulds (5 ​mm ​× ​3 ​mm for *in vitro* analysis and 7 ​mm ​× ​2 ​mm for the preclinical study) and lyophilized to create a scaffold which was then subjected to dehydrothermal treatment (115 ​°C, under vacuum for 24 ​h). The dehydrothermal treatment was performed to both physically cross-link the scaffolds and sterilize the scaffolds. The resulting scaffolds were predominantly collagenous in nature, with the majority of sulfated glycosaminoglycan (sGAG) and DNA removed during scaffold fabrication. The scaffolds contained AC-ECM at a concentration of 10 ​mg/ml as this concentration was previously found to be optimal for supporting chondrogenic differentiation of human stromal cells derived from infrapatellar fat pad tissue [28]. For TGF-β3 loaded scaffolds, 300 ​ng of TGF-β3 (PeproTech) diluted in PBS was soak loaded dropwise onto the scaffolds 2 ​h before use. 300 ​ng was selected as the dose of TGF-β3 in order to provide a dose of TGF-β3 which was equivalent to the TGF-β3 dose during *in vitro* studies. In the *in vitro* studies, TGF-β3 was provided at a concentration of 10 ​ng/ml; each scaffold was in 2.5 ​ml of media which would have been replenished 12 times over the course of the study.

Ligament ECM (LIG) control scaffolds were fabricated using the same basic procedure as described above. ECM was harvested from the anterior cruciate ligaments of pigs, solubilised with pepsin and the type I collagen was preferentially extracted from the sample using NaCl at a final concentration of 0.8 ​M [38,39]. ECM and glyoxal cross-linking concentrations were matched to the AC-ECM scaffolds (10 ​mg/ml ECM with 5 ​mM Glyoxal).

### 2.3 Effect of ECM source on BM-MSC gene expression

To examine the effect of ECM in a 2D system, pepsin solubilised AC or LIG-ECM at a concentration of 0.05 ​mg/ml was coated onto 6 well plates (Nunc). ECM was allowed to bind to the tissue culture plastic for 2 ​h before rinsing with PBS (Gibco). 2.5 ​× ​10⁵ human bone marrow derived mesenchymal stromal cells (BM-MSCs - Lonza) were then seeded into each well on the ECM substrates or into untreated control well. Cells were then incubated in expansion media (DMEM with 10% FBS and 1% penicillin-streptomycin (all Gibco)) for 48 ​h before RNA isolation. To examine the effect of ECM in a 3D system, 1 ​× ​10⁶ BM-MSCs were seeded onto scaffolds. BM-MSCs were allowed to attach to the scaffolds for 1 ​h in an incubator at 37 ​°C before addition of expansion media. Cells were cultured for 48 ​h before RNA isolation. RNA isolation was performed using a RNeasy Mini Kit (Qiagen) according to the manufacturer's instructions. cDNA was generated using a high capacity cDNA reverse transcription kit (Fisher) according to manufacturer's protocol. Gene expression was analysed using KiCqStart® SYBR® Green Primers for *Col1a1, Col2a1, SOX9* and *GAPDH* (Sigma). QPCR was performed on a StepOnePlus instrument (Applied Biosystems) to obtain comparative ΔΔCt values. Data was normalized to *GAPDH.*

### 2.4 Effect of ECM source on BM-MSC micromass pellet differentiation

2.5 ​× ​10⁵ BM-MSCs were cultured in a micromass pellet system as previously described [40,41]. Pellets were cultured either in a growth factor free chondrogenic differentiation medium (CDM-) which consisted of high glucose DMEM supplemented with 1% penicillin-streptomycin, 100 ​μg/ml sodium pyruvate (Sigma), 40 ​μg/ml l-Proline (Sigma), 50 ​μg/ml l-ascorbic acid-2-phosphate (Sigma), 1.5 ​mg/ml bovine serum albumin (BSA-Sigma), 1× insulin transferrin selenium (ITS- Gibco), 100 ​nM dexamethasone (Sigma) for 28 days. This media was supplemented with 0.05 ​mg/ml of solubilised AC-ECM or LIG-ECM during twice weekly media changes. As a positive control, pellets were also cultured in CDM ​+ ​that consists of the CDM formulation above plus 10 ​ng/ml transforming growth factor beta-3 (TGF-β3 –PeproTech). sGAG quantification was performed using a 1, 9 dimethylmethylene blue (DMMB) assay according to the manufactures protocol (Blyscan sulfated sGAG assay kit, Biocolor).

### 2.5 Seeding ECM scaffolds

1 ​× ​10⁶ human bone marrow derived mesenchymal stromal cells (BM-MSCs - Lonza) were seeded dropwise onto individual 5 ​× ​3mm scaffolds suspended in 25 ​μL of expansion media (DMEM with 10% FBS and 1% penicillin-streptomycin). Scaffolds were placed into individual wells of a 12 well plate (Nunc). BM-MSCs were allowed to attach to the scaffolds for 1 ​h in an incubator at 37 ​°C. After BM-MSC attachment, 2.5 ​ml of CDM plus 10 ​ng/ml TGF-β3 was added to each well. The BM-MSC seeded constructs were maintained in CDM for 28 days with the CDM ​+ ​TGF-β3 being replenished three times per week.

### 2.6 In vitro study analysis

BM-MSC seeded constructs (Day 28) were analysed for DNA and sGAG content. Prior to performing assays, constructs were enzymatically digested with papain (125 ​μg/ml - Sigma) in a buffer containing 100 ​mM Sodium Phosphate (Sigma) with 5 ​mM Na_2_EDTA (Sigma) at pH 6.5 as previously described [42]. sGAG quantification was performed using a DMMB assay as described previously. Quantification of dsDNA in the digested constructs was performed using a Quant-iT Pico Green dsDNA kit (Invitrogen) according to the manufactures protocol. Calcium content of constructs was determined by a calcium liquid colormetric assay as per the kit manufactures instructions (Sentinel Diagnostics). For histological analysis, samples were fixed with 4% paraformaldehyde (Sigma). Samples were dehydrated and wax embedded. Embedded constructs were then sectioned at a thickness of 5 ​μm using a microtome. Sections were stained with 1% Alcian blue to examine sulfated glycosaminoglycan (sGAG) and picrosirius red to examine collagen deposition. To identify the specific collagen types present in the constructs, immunohistochemistry was performed to detect collagen type I (Abcam ab90395 1:400), collagen type II (Santa Cruz-sc52658 1:400), and collagen type X (Abcam ab49945 1:200) as previously described [28].

### 2.7 TGF-β3 migration assay

To determine the ability of BM-MSCs to migrate into AC-ECM scaffolds, we established a culture system to mimic the dimensions of the defect in the preclinical model. A 4% agarose (Sigma) female mould was cast to produce an empty cylinder of 6 ​mm Ø and 6 ​mm height. AC-ECM scaffolds were then either soak loaded dropwise with PBS (-TGF-β3) or with PBS containing 300 ​ng of TGF-β3 (+TGF-β3). Scaffolds were then incubated for 2 ​h in an incubator at 37 ​°C. The scaffolds were then placed into the base of the cylindrical agarose mould. An impermeable agarose disk (6 ​mm Ø by 1 ​mm height) was placed on top of the scaffold. To mimic the preclinical study, the same microfracture drilling strategy was employed. A surgical Kirschner wire (gSource) was used to pierce circular holes in the agarose disk. Five holes were pierced in total, one central hole (1.6 ​mm Ø) and four holes (0.7 ​mm Ø) at 12, 3, 6 and 9 o'clock to the central hole. Next, 2% Fibrin hydrogels were fabricated as previously described [43]. Fibrin gels (6 ​mm Ø by 2 ​mm height) contained BM-MSCs at a concentration of 20 ​× ​10⁶ cells/ml. These cell-seeded fibrin gels were then placed on top of the agarose disk, the assembled culture system was them transferred to a 12 well tissue culture plate and incubated with expansion media for 7 days after which the DNA content in the AC-ECM scaffolds was quantified by a Quant-iT Pico Green dsDNA assay (Invitrogen).

### 2.8 Surgical procedure

**Fig. 1:**
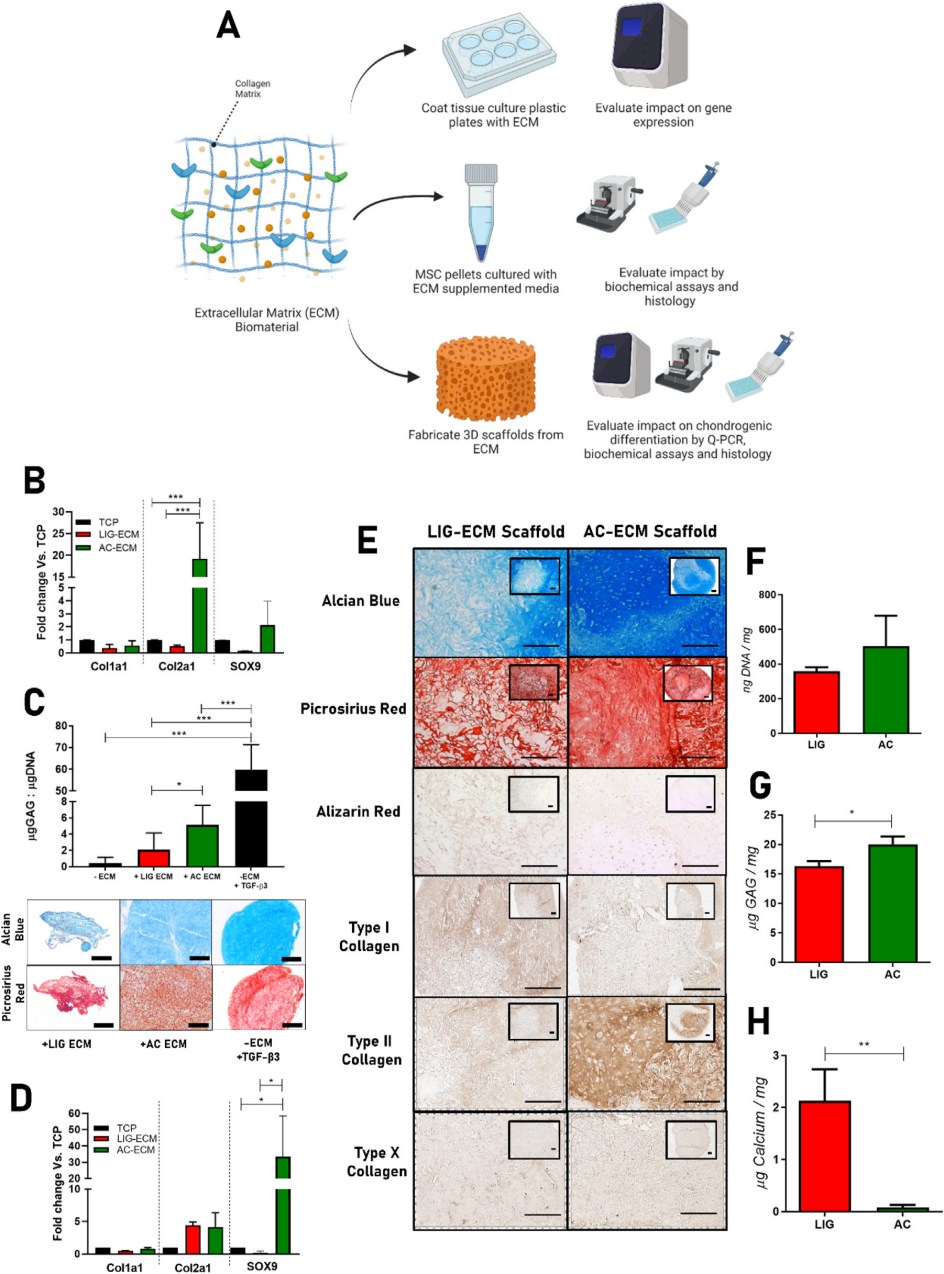
Evaluation of impact of ECM biomaterials on MSC differentiation and gene expression. Schematic diagram detailing analysis workflow **(A).** Gene expression profile of BM-MSCs on cultured in a monolayer on TCP, LIG-ECM or AC-ECM coated substrates after 48 ​h was analysed by Q-PCR **(B)**. 250,000 BM-MSCs were cultured in a micromass pellet assay for 28 days and supplemented with either LIG or AC-ECM, sGAG deposition was analysed by DMMB assay and by Alcian Blue and Picrosirius Red histological staining **(C)**. Gene expression profile of BM-MSCs cultured on TCP or LIG-ECM and AC-ECM 3D scaffolds after 48 ​h was analysed by Q-PCR **(D).** GAG deposition (Alcian Blue), collagen deposition (Picrosirius Red) and mineralisation (Alizarin Red) and type I/II/X collagen deposition was evaluated by histological/immunohistochemical staining of day 28 BM-MSC seeded *in vitro* scaffold constructs. **(E)**. Biochemical quantification of DNA **(F)**, GAG **(G)** and Calcium **(H)** was performed on day 28 constructs. All error bars denote standard deviation, ∗p<0.05, ∗∗p<0.01, *n*=4-6. Scale bars = 200μm.

Surgery was performed on skeletally mature female Saanen goats (age at start of study ​= ​4.29 years ​± ​0.27). The mean weight of the animals was 67.8 ​kg (±9.9 ​kg). Goats were sedated using diazepam (0.2 ​mg/kg IV) and butorphanol (0.2 ​mg/kg IV). An epidural was administered using morphine (0.2 ​mg/kg) and lidocaine HCL (1 ​mg/kg). Anesthesia was induced with propofol on effect (max. dose 6 ​mg/kg IV) and maintained with isoflurane. Goats were placed in dorsal recumbency and an arthrotomy of each stifle joint was then performed using the lateral para-patellar approach. Bi-lateral surgery was performed on all animals. 6 ​mm Ø by 1 ​mm deep chondral defects were created in the medial femoral condyles using a 6 ​mm biopsy punch to mark the defect diameter followed by cartilage removal using a curette. MFX was performed in all defects using a Kirschner wire (1.6 ​mm Ø for the central hole and 0.7 ​mm Ø for all other holes [gSource]). AC-ECM scaffolds were maintained in position using a custom made, biodegradable, 3D printed fixation device fabricated using polycaprolactone (PCL, Perstop). 3D printed fixation devices were only implanted into animals receiving a scaffold. The shaft of the fixation device was fabricated to be the slightly smaller than the central MFX hole (1.58 ​mm Ø) so that the device and scaffold could be push-fit into the defect. The diameter of the flexible head/membrane (single fibre of PCL) of the fixation device holding the scaffold in position was 5.8 ​mm (Fig. 1). The stifle joints were randomized and assigned to one of the three treatment groups: 1) Microfracture only, 2) MFX ​+ ​AC-ECM scaffold, 3) MFX ​+ ​TGFβ3 loaded AC-ECM scaffold. Following routine closure of the joint capsule, subcutaneous tissues and skin with sutures, Carprofen (1.4 ​mg/kg) and morphine (0.2 ​mg/kg) both subcutaneously were administered for analgesia. Following surgery, goats were housed in indoor pens and were allowed full weight bearing immediately. NSAIDs (carprofen 1.4 ​mg/kg) and antibiotics (Amoxicillin plus clavulanic acid 8.75 ​mg/kg) were administered for 5 days post-surgery. Two weeks post-operatively, following removal of sutures, animals were let out to pasture for the remainder of the study period. Tissue repair was evaluated at 6 months post-surgery.

### 2.9 Evaluation of repair tissue

1.5 ​cm^3^ sections containing the defect site were harvested from the goats using an oscillating bone saw. Before fixation, gross morphological images were taken with a digital microscope system (Ash Inspex HD 1080p) for macroscopic evaluation. This macroscopic scoring system has a maximum score of 8 and evaluates the edge integration of the scaffold with the native tissue, the smoothness of the cartilage surface, the degree of defect filling and the colour/opacity of the neocartilage in the defect.

For histological analysis; samples were fixed in 10% neutral buffered formalin ​solution (Sigma) for 72 ​h with agitation. Samples were then decalcified with Decalcifying solution lite (Sigma) until all mineral was removed, which was confirmed by x-ray analysis. Next, the defect blocks were manually cut across the defect site with a scalpel. Due to the presence of the shaft of the pin and the manual slicing, the result we obtain is two blocks of slightly different size, with one containing the shaft of the pin which is still intact. We then performed histology on the section without the residual pin. It should therefore be noted that the histological images that are presented are not from the exact center of the defect site but are approximately 800 ​μm to 1 ​mm from the central MFX hole.

Demineralized wax-embedded constructs were sectioned at 10 ​μm and stained with safranin O and picrosirius red. Histological scoring was performed using a modified ICRS II scoring system [44]. Safranin O staining was also used in combination with Photoshop CS6 to quantify the area of positively stained cartilage within the defect site. Picrosirius red stained samples were imaged under polarized light microscopy to investigate collagen fibre orientation. The Directionality plugin for ImageJ [45] was used to quantify the mean orientation and angular dispersion of the collagen fibres observed in the superficial and deep zones of the regenerated AC. Immunohistochemistry was performed for collagen type II (Santa Cruz-sc52658 1:400), collagen type I (Abcam ab90395 1:400) as previously described [28]. For the detection of Lubricin, antigen retrieval was performed using Chondroitinase ABC (0.25 units/ml - Sigma) for 60 ​min at 37 ​°C. Non-specific binding was blocked using a solution of 1% bovine serum albumin (Sigma) and 10% donkey serum (Sigma) in PBS for 60 ​min. *Anti*-lubricin primary antibody (Millipore MABT400 1:500 dilution) was then incubated on the samples overnight at 4 ​°C. Endo-peroxidase activity was quenched using 3% hydrogen peroxide solution (Sigma). Next, the secondary antibody (*Anti*-IgG mouse (Sigma B7151, 1.5:200 dilution) was incubated on the samples for 60 ​min at room temperature. Staining was developed using a DAB (3,3′-Diaminobenzidine) substrate kit (Vector Labs). For reference, positive staining of native goat AC samples and negative control (no primary) images are provided in Supplementary Fig. 3. To quantify the extent of positive type II collagen staining in the defect site defect, a 2 ​× ​6 ​mm region of interest central to the defect was selected and DAB positive staining was quantified using the plugin IHC profiler for ImageJ.

### 2.10 Statistical analysis

Results are presented as mean±standard deviation. Statistical analysis was performed using Graph Pad Prism 8 (San Diego, USA). Statistical differences were analysed by Kruskal–Wallis one-way analysis of variance (ANOVA) for multiple comparisons to compare experimental conditions or in the case of comparing two experiment groups a paired or unpaired *t*-test was used when appropriate. Statistically significant changes are marked as ∗ ​= ​p<0.05; ∗∗ ​= ​p ​≤ ​0.01; ∗∗∗ ​= ​p ​≤ ​0.001.

## 3 Results

### 3.1 Development and *in vitro* evaluation of a novel articular cartilage repair system

We have previously demonstrated that solubilised AC-ECM derived scaffolds elicit a minimal immune response and support the robust chondrogenic differentiation of infrapatellar fat pad derived stomal cells *in vitro* [28]. However, whether solubilised AC-ECM is inherently chondro-inductive (i.e. capable of inducing chondrogenesis in the absence of exogenous growth factors) and superior to type I collagen-based biomaterials in supporting chondrogenesis remains unclear. To answer this question, we first cultured human bone marrow stromal cells (MSCs) on the surface of tissue culture plastic (TCP) coated with solubilised porcine AC-ECM and ligament (LIG)-ECM and examined gene expression after 48 ​h. In the absence of exogenous chondrogenic factors, MSCs cultured on AC-ECM expressed significantly higher levels of type II collagen (*Col2a1*) gene expression than those cultured on either uncoated TCP or LIG-ECM, with no differences in the expression of type I collagen (*Col1a1*). A trend towards an upregulation in SRY-box transcription factor 9 (*SOX9*), a key chondrogenic transcription factor was also observed in MSCs cultured on AC-ECM coated plates (Fig. 1B). Furthermore, in a pellet culture model designed to promote cellular condensation, MSCs (without the addition of exogenous growth factors) secreted a cartilaginous matrix when stimulated with solubilised AC-ECM, depositing significantly higher levels of sGAGs than cells stimulated with solubilised LIG-ECM, although not to the same level as cells stimulated exogenously with the chondrogenic factor TGF-β3 (Fig. 1C). When GAG and collagen deposition was examined histologically, we observed that pellets supplemented with the addition of LIG-ECM were smaller in size, failed to become spherical and were relatively fibrous in nature when compared to other groups (Fig. 1C). Together this data demonstrates that solubilised AC-ECM is inherently more chondro-inductive than solubilised LIG-ECM.

With a view to utilizing such biomaterials to augment the outcomes of marrow stimulation techniques such as MFX, we next assessed the capacity of porous three-dimensional AC-ECM scaffolds to support chondrogenesis of human bone marrow stromal cells (MSCs) *in vitro.* To examine early changes in gene expression, we cultured the MSC-seeded scaffolds and harvested RNA after 48 ​h. In the absence of exogenous chondrogenic factors, MSCs cultured on AC-ECM scaffolds expressed significantly higher levels of *SOX9* gene expression (30-fold) than those cultured in LIG-ECM scaffolds, with no significant differences in the expression of type I or type II collagen observed at this timepoint (Fig. 1E).

Compared to ECM derived scaffolds fabricated from type I collagen rich LIG-ECM, scaffolds derived from type II collagen rich AC-ECM supported higher levels of sulfated glycosaminoglycan (sGAG) deposition as demonstrated by alcian blue histological staining (Fig. 1E) and biochemical quantification (Fig. 1G) after 28 days of *in vitro* culture. AC-ECM scaffolds supported the development of a cartilage tissue rich in type II collagen (Fig. 1E), with less intense staining for this cartilage-specific marker observed in tissues engineered within LIG-ECM scaffolds. Furthermore, neither the LIG-ECM or AC-ECM scaffolds seemed to promote an endochondral phenotype as they stained weakly/negatively for types I and X collagen and alizarin red (for calcium deposits), however when calcium content was quantified biochemically, the LIG-ECM scaffolds were found to support higher levels of calcium deposition than the AC-ECM scaffolds (Fig. 1F).

**Fig. 2:**
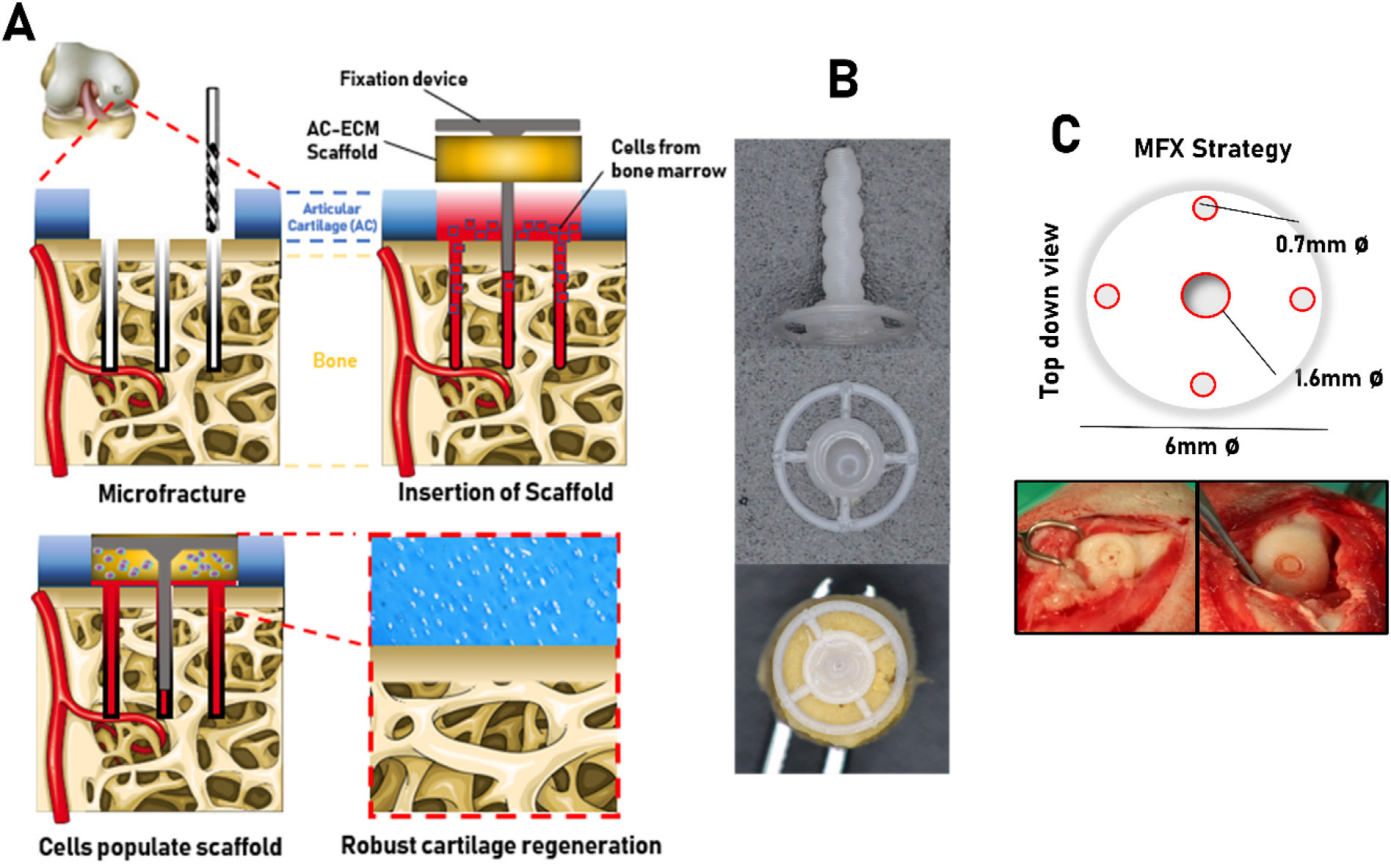
Development of fixation system. Schematic diagram illustrating the clinical workflow for the system **(A)**. Stereoscopic micrographs of the 3D printed fixation system detailing the ridges on the shaft and mounting of the AC-ECM scaffold **(B)**. Schematic representation and *in situ* photographs of MFX drilling strategy used during animal experiments **(C)**. Evaluation of cartilage repair in a clinically relevant large animal model.

In order to concurrently promote robust cartilage regeneration and fixate such a scaffold within an articular cartilage defect site, a novel AC repair system was developed that encompasses an AC-ECM scaffold coupled with a 3D printed fixation device. This device was designed to be compatible with current MFX or micro-drilling techniques for AC repair. The proposed clinical workflow is illustrated in Fig. 2A. 3D printed fixation devices were fabricated by fused deposition modelling of polycaprolactone (PCL), a biodegradable polymer that is used in existing FDA approved medical devices (Fig. 2B). The fixation devices were designed so that the shaft of the pin had a slightly smaller diameter (1.58 ​mm Ø) than the central MFX hole (1.60 ​mm Ø) to allow the scaffold to be quickly and easily push-fit into the defect by the surgeon. A schematic of the MFX drilling strategy and photographs of the device *in situ* are provided in Fig. 2C. Pilot studies confirmed that the pin remained in place 2 weeks and 1 month after surgical placement of the device within AC defects created with the medial femoral condyle of goats (see Supplementary Fig. 1).

**Fig. 3:**
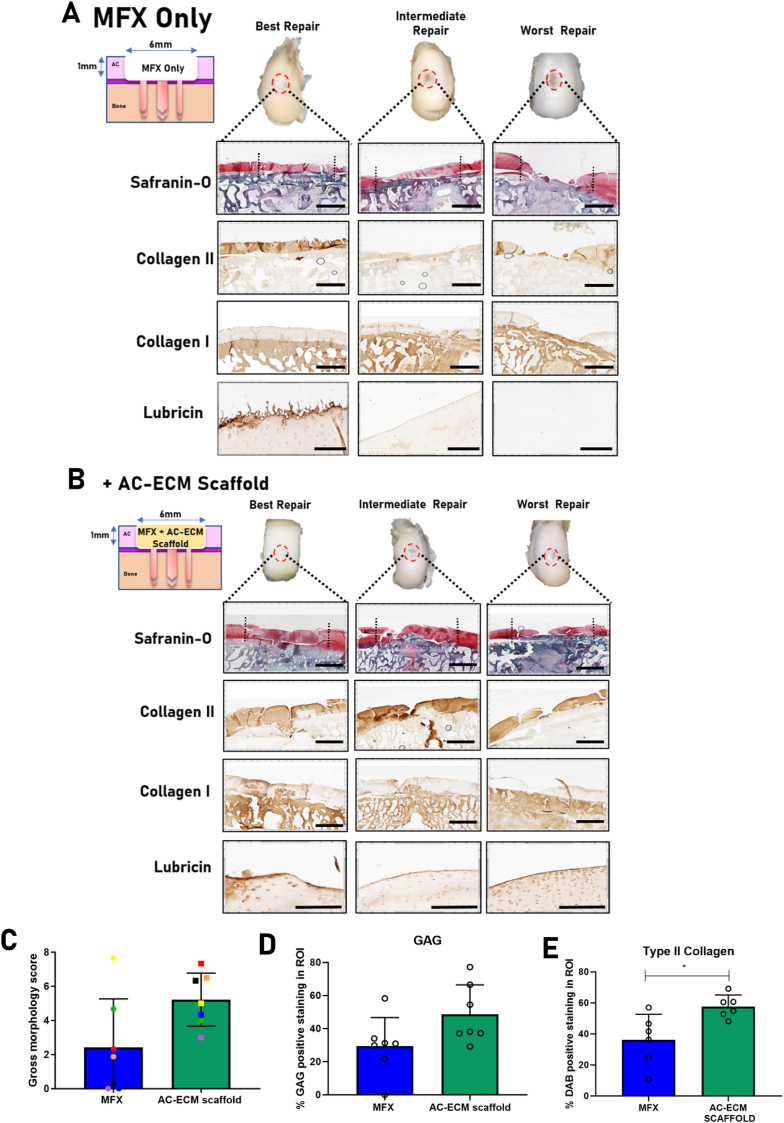
Evaluation of cartilage repair system in preclinical goat model. 6 months after implantation, the defect sites were excised, imaged and processed for histology. MFX only treated animals exhibited high variation in defect fill and repair quality **(A)**. AC-ECM scaffold assisted MFX animals demonstrated improved and more consistent repair quality when compared to MFX only controls **(B)**. Blinded macroscopic scoring was used to quantify visual repair of the defects **(C)**. GAG **(D)** and type II collagen **(E)** Deposition within the defect region of interest was quantified using image analysis software. All error bars denote standard deviation, ∗p<0.05, *n*=7 animals. Scale bar =2mm in Saf-O, Col I and Col II and 200μm for Lubricin IHC. Black dashed lines indicate defect borders. Incorporation of TGFβ-3 within AC-ECM scaffolds enhances MSC recruitment in vitro and endogenous cartilage repair in vivo

Having demonstrated *in vitro* that AC-ECM scaffolds support robust chondrogenesis of MSCs with no aberrant calcification, we next sought to test the capacity of these scaffolds to improve the outcomes of the MFX procedure in a clinically relevant caprine model of articular cartilage repair. To this end, 6 ​× ​1mm chondral defects were created using a curette in the medial femoral condyles of both hind limbs in skeletally mature goats. The two hind limbs of each animal were randomly assigned either MFX only or MFX coupled with scaffold implantation. Scaffolds were maintained in position in the defects using the 3D printed fixation device. Animals were euthanized after 6 months and the quality of tissue repair was evaluated macroscopically and histologically. We observed that in the MFX only group the quality of the repair was highly variable; while some animals were found to have high levels of defect fill, with the repair tissue staining positive for sGAG and type II collagen deposition, approximately half of the animals examined were found to have poor levels of defect fill. In addition, the repair tissue in these animals stained weakly or negatively for sGAG, type II collagen and lubricin deposition, key makers of stable hyaline-like cartilage tissue (Fig. 3A). In defects treated with AC-ECM scaffold assisted MFX, we observed more consistent levels of high-quality cartilage repair tissue. Macroscopically, superior defect fill was observed in scaffold treated defects compared to MFX only controls. For the majority of animals, the repair tissue stained positive for sGAG and type II collagen, and superficially for lubricin, indicating the production of hyaline-like cartilage by the host cells that infiltrated the scaffold post-implantation (Fig. 3B). Upon macroscopic grading of the defects by blinded reviewers, we observed that on average the MFX only group scored 2.4 points, whereas the AC-ECM scaffold group had a mean score of 5.2 points out of a maximum of 8 (p ​= ​0.06; Fig. 3C). Details of the scoring system [46,47], characteristics examined, and score values can be found in Table 1. For comparison purposes, histological images from historical control animals have been provided in Supplementary Fig. 3. Quantification of both sGAG and type II collagen deposition from all animals was performed using image analysis software. Upon quantification, a trend towards increasing sGAG deposition was observed in the AC-ECM scaffold treated animals when compared to MFX only controls (MFX - 29% of region of interest (ROI) stained positive; AC-ECM – 50% of ROI stained positive; p ​= ​0.09; Fig. 3D). Significantly higher levels of type II collagen deposition were observed in scaffold treated defects after 6 months *in vivo* (MFX – 36% of ROI stained positive; AC-ECM – 58% of ROI stained positive; p ​= ​0.016; Fig. 3E). Interestingly, scaffold implantation appeared to have the most benefit in animals that responded poorly to MFX treatment alone, as evident by comparing healing outcomes in animals with poor outcomes in response to MFX (represented by the purple, black and blue symbols in Fig. 3C) with corresponding outcomes in the opposite limb treated using scaffold assisted MFX. No adverse events related to the surgery or device were observed over the 6 months. While these results were highly promising, hyaline cartilage repair was not consistently observed in response to scaffold implantation. To address this concern, we next sought to establish if functionalization of AC-ECM derived scaffolds with a known chemotactic growth factor would further enhance the quality and consistency of AC repair.

**Table 1:** Gross morphology scoring system used for repair evaluation. Max score ​= ​8.

Characteristic	Grading	Score
Edge integration (new tissue relative to native cartilage)	Full	2
	Partial	1
	None	0
Smoothness of cartilage surface	Smooth	2
	Intermediate	1
	Rough	0
Cartilage surface, degree of filling	Flush	2
	Slight depression	1
	Depressed/overgrown	0
Colour of cartilage, opacity or translucency of the neocartilage	Opaque	2
	Translucent	1
	Transparent	0

**Fig. 4:**
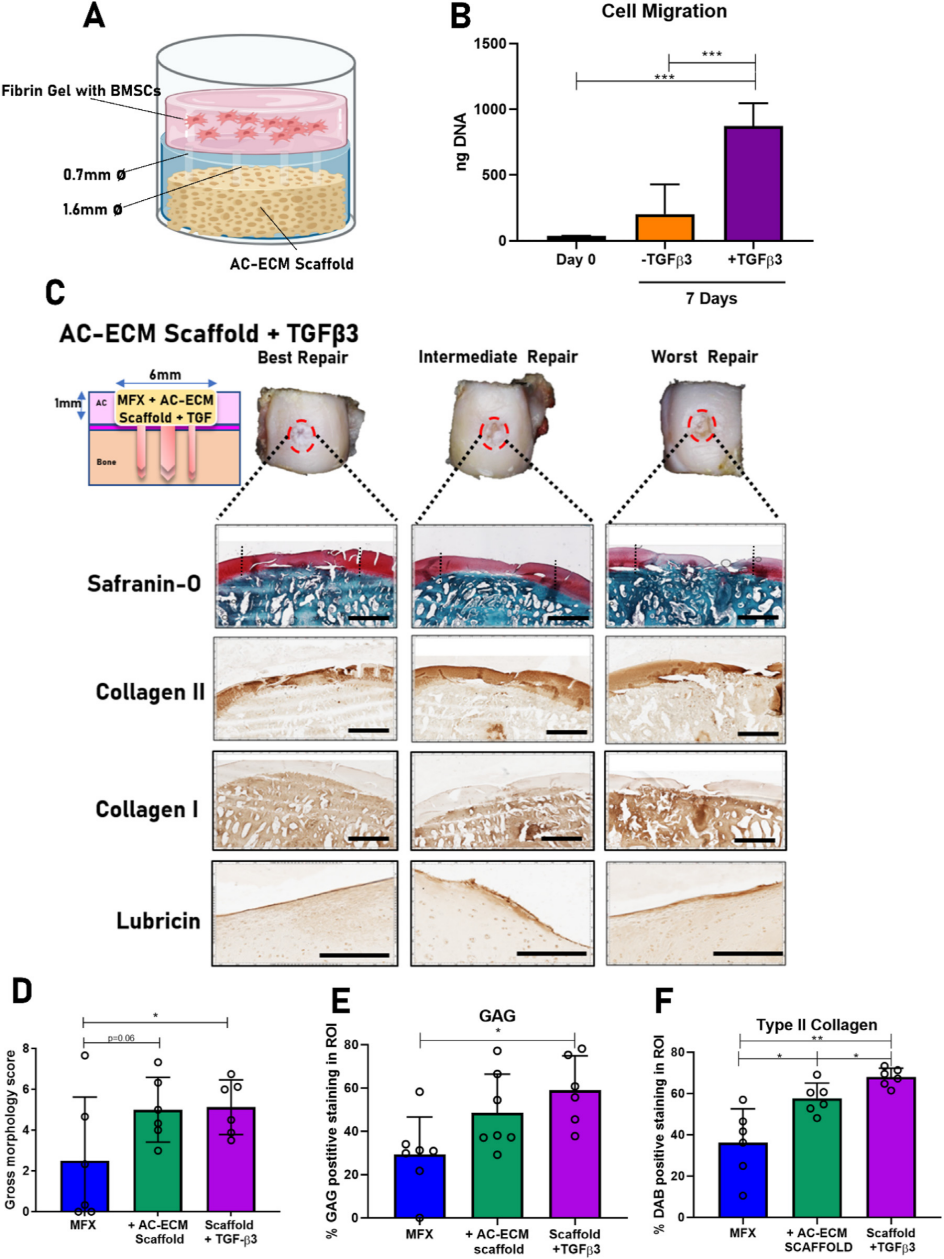
Incorporation of TGF-β3 with AC-ECM scaffolds enhances MSC migration *in vitro* and endogenous cartilage repair in a preclinical goat model. A schematic diagram of the migration assay employed to assess MSC migration **(A)**. DNA assay to quantify the migration of the MSCs into the AC-ECM scaffold after 7 days in culture (*n*=*4)***(B)**. 6 months after implantation, the defect sites were excised, imaged and processed for histology. MFX only treated animals exhibited high variation in defect fill and repair quality. AC-ECM scaffold ​+ ​TGF-β3 animals demonstrated improved and more consistent repair quality when compared to MFX only controls in Fig. 2**(C)**. Blinded macroscopic scoring was used to quantify visual repair of the defects **(D)**. GAG **(E)** and type II collagen.**(F)** deposition within the defect region of interest was quantified using image analysis software. All error bars denote standard deviation, ∗p<0.05, ∗∗ p<0.1 *n*=6-7 animals. Scale bar =2mm in Saf-O, Col I and Col II and 200μm for Lubricin IHC. Black dashed lines indicate defect borders. ECM scaffolds promote recapitulation of the native collagen fibre alignment in the superficial zone of the repair tissue

The use of TGF-β to promote chondrogenic differentiation of MSCs has long been established [40]. Members of the TGF-β superfamily have also been shown to act as potent chemotactic factors [48,49], with TGF-β3 shown to enhance endogenous bone [50] and cartilage repair [51]. Using an *in vitro* model of AC repair, we next sought to assess whether loading of AC-ECM derived scaffolds with TGF-β3 would enhance the capacity of the construct to recruit MSCs from the local environment. To mimic the *in vivo* scenario as closely as possible, we developed a “chondral defect in a dish” migration assay, where the ECM scaffold was placed on top of a fibrin gel containing MSCs (Fig. 4A). After 7 days *in vitro*, significantly more (4-fold) MSCs had migrated from the cell loaded fibrin hydrogel into the TGF-β3 loaded scaffold compared to the TGF-β3 free control scaffold (Fig. 4B).

Having established that scaffold functionalization with TGF-β3 promotes the recruitment of MSCs, we next sought to evaluate if these growth factor loaded scaffolds would further enhance cartilage repair *in vivo*. Cartilage defect repair following implantation of these growth factor eluting constructs was evaluated 6 months post-surgery and compared to the outcomes observed previously using MFX only and scaffold assisted MFX (no growth factor). No adverse events related to the surgery or device were observed over the 6 months. Consistent hyaline-like cartilage repair, with reduced levels of variability, was observed in animals treated with TGF-β3 eluting scaffolds. This improvement in repair was observed both macroscopically and histologically. Histologically the repair tissue consistently stained positively for sGAG and type II collagen, with distinct positive lubricin staining observed in the superficial zone of all defects (Fig. 4C). The repair tissue of all defects also stained negatively for type I collagen (Fig. 4C). Blinded macroscopic scoring revealed significantly greater scores in the AC-ECM scaffold ​+ ​TGF-β3 (5.1 points) group when compared to MFX only group (2.4 points) (Fig. 4D). Significantly more sGAG was found to be deposited in the defect site in the +TGF-β3 cohort versus MFX only control animals. Animals treated with MFX only were found to have GAG positive tissue in 29% of the region of interest compared to 59% in the AC-ECM scaffold ​+ ​TGF-β3 group (Fig. 4E). The AC-ECM scaffold ​+ ​TGF-β3 cohort was also found to promote significantly higher levels of type II collagen deposition compared to both the MFX only cohort and the unloaded AC-ECM scaffold (Fig. 4F).

**Fig. 5:**
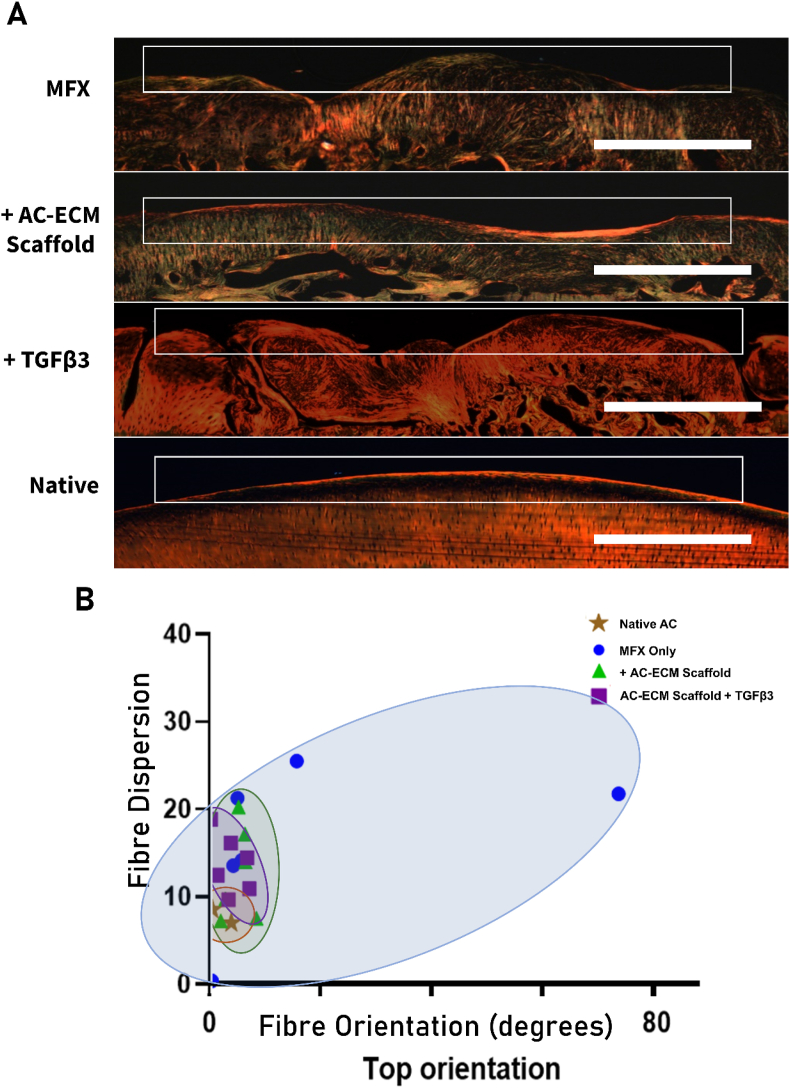
AC-ECM scaffolds promote the recapitulation of native collagen fibre alignment in the superficial zone. Histological sections were stained with picrosirius red and then imaged using polarised light microscopy. Scale bar ​= ​1 ​mm **(A)**. The orientation and dispersion of the collagen fibre orientation of the superficial zone of the defect site relative to native controls was plotted. A lower dispersion value indicates higher consistency within the region of interest. Collagen fibres that run parallel to the articulating surface have an orientation of 0°, whereas fibres that are perpendicular to the surface have an orientation of 90° in native caprine cartilage tissue. **(B)**. *n* ​= ​6–7 animals.

Having observed distinct lubricin staining in the superficial zone of the repair tissue in both the AC-ECM scaffold and AC-ECM scaffold ​+ ​TGF-β3 groups, indicating some recapitulation of the native zonal nature of AC, we were motivated to examine the collagen fibre alignment within the repair tissue in more detail. Following picrosirius red staining of histological samples, the samples were subjected to polarised light microscopy (PLM) to visually determine the collagen fibre orientation in the superficial zone (Fig. 5A). The predominant angle of orientation and the dispersion of the collagen fibre orientation was quantified in the superficial zone using the imageJ plugin *Directionality* [29,45]*.*Previous work has shown that native caprine cartilage has a parallel fibre orientation relative to the superficial zone, with the majority of fibres having an orientation approaching 0° and a low level of dispersion [29]. Here we demonstrate that MFX only treated animals have a high level of fibre dispersion, as well as an average fibre orientation angle that noticeably deviates from that observed in normal cartilage (mean 9.9° ​± ​25.48°), as demonstrated by the blue eclipse. However, when animals are treated with the AC-ECM scaffold, either with or without TGF-β3 supplementation, both the average angle of collagen fibre orientation and the levels of dispersion was seen to approach native values, as represented by the green and purple ellipses respectively (Fig. 5B). Upon quantification the AC-ECM scaffold group had a mean fibre orientation angle of 5.29° (±2.19) and the AC-ECM scaffold ​+ ​TGF-β3 had a mean fibre orientation angle of 3.98° (±2.52°)

## 4 Discussion

The overall goal of this study was to develop a cell-free, single-stage, ‘off-the-shelf’ device capable of enhancing endogenous AC repair. To this end we coupled an articular cartilage ECM derived scaffold, which is inherently chondro-inductive and capable of supporting robust chondrogenic differentiation of MSCs *in vitro*, with a biodegradable fixation device that could quickly and securely maintain the implant within a cartilage defect *in vivo*. Having developed the device, we then tested the ability of the construct to augment and improve the current surgical standard of care for chondral defects (microfracture) in a clinically relevant caprine model. In our preclinical caprine model, the AC-ECM derived scaffold improved tissue repair outcomes when compared with MFX only. We also demonstrated that the loading of such ECM scaffolds with a known chemotactic growth factor, TGF-β3, increased MSC recruitment *in vitro* and enhanced the consistency and quality of the AC repair *in vivo*.

Collagen-based biomaterials currently in clinical use for cartilage repair are predominantly made of type I collagen [24]. For example, the MACI [15], NeoCart® [52], Novocart-3D® [53], MaioRegen [54] and CaRes® [55] scaffolds are all based on type I collagen. While type I collagen is both ubiquitous and the major structural protein present in many tissues, it is not found abundantly in hyaline cartilage, where type II collagen makes up over 80% of the total collagen network [56]. Type I collagen, while being biocompatible, lacks specific, biochemical chondrogenic cues to drive endogenous cartilage regeneration. This fact motivated the use of an AC-ECM derived scaffold as the basis of a novel device to enhance the endogenous regeneration of chondral defects. *In vitro,* we first demonstrated that unlike solubilised LIG-ECM, AC-ECM is inherently chondro-inductive, and in the absence of exogenous growth factors instructs MSCs to increase the expression of cartilage-specific genes and secrete a matrix rich in sGAGs. Furthermore, porous scaffolds generated from solubilised AC-ECM supported the development of a cartilaginous tissue that was rich in sGAG and type II collagen, with a reduced hypertrophic profile compared to tissues engineered using scaffolds generated from LIG-ECM. While further studies are required to fully elucidate the mechanism of action behind the pro-chondrogenic effects of the AC-ECM, we have previously observed that when human macrophages are seeded onto such scaffolds that they upregulate expression of interleukin-8 (IL-8) and basic fibroblast growth factor (bFGF), both of which have been demonstrated to be pro-chondrogenic [[57], [58], [59]]. In addition, AC-ECM scaffolds are predominately composed of type II collagen, which itself has been shown to promote chondrogenic differentiation in the absence of growth factors [25].

Cartilage ECM scaffolds and tissue engineered cartilage templates have previously been used to induce endochondral bone formation *in vivo* as a developmentally inspired route to bone formation [[60], [61], [62]]. While small levels of calcium accumulation were observed in the cartilage tissues engineered *in vitro* within the LIG-ECM scaffolds (albeit substantially lower to previous work from our group [63] which fabricated osteoinductive ECM scaffolds from growth plate cartilage), the AC-ECM scaffolds were able to fully supress both calcium and type X collagen deposition. Previously, when BM-MSCs were seeded onto AC-ECM scaffolds and cultured in osteogenic media, we observed complete inhibition of osteogenic differentiation with low levels of chondrogenic differentiation despite the potent osteogenic stimuli provided by the media [57]. This is crucial as bone marrow stromal cells have a proclivity to undergo hypertrophic differentiation *in vitro* and *in vivo*, with aberrant ossification known to be deleterious in a cartilage repair setting [41]. In addition to appearing to suppress this endochondral phenotype *in vitro*, in a subsequent study, when AC-ECM scaffolds were seeded with BM-MSCs and implanted in a mouse subcutaneous model for 8 weeks, only minimal mineral deposition was observed [64]. Here we also observed limited evidence for calcification of the repair tissue *in vivo* following implantation of the AC-ECM scaffolds into goat chondral defects. No evidence of osteophytes were observed. Similar results were observed when these scaffolds were loaded with TGF-β3, which generally promoted the development of a cartilage repair tissue that stained negatively for type I collagen, which mitigates concerns that intra-articular delivery of such proteins might increase the risk of cartilage calcification.

Biomaterial implants must remain stable and fixed within the chondral defect in order to promote functional restoration of damaged AC tissue. The two most common fixation methods used clinically are sutures and glues [24]. Currently, sutures offer robust fixation of scaffolds but in addition to prolonging surgical time, suturing has been shown to induce osteoarthritis-like changes in the adjacent cartilage [65]. While glues comprising of fibrin are currently in clinical use due to their biocompatibility and ease of use, the biomechanical loads required to induce failure were found to be significantly less than sutures [34,66]. In addition, fibrin glues have been shown to inhibit endogenous cell migration [67]. Subchondral bone suture anchors can also be employed to improve fixation, however this technique still requires suturing of the scaffold to the anchor which is time-consuming and potentially damaging to the scaffold [68]. Rigid pin fixation using absorbable and non-absorbable devices of scaffolds and gels has been performed with varying levels of success [34,36,37]. After the MFX procedure, our cartilage repair device can be push-fit into the MFX hole in a matter of seconds, with the fixation system easily and quickly securing scaffolds into the base of chondral defects. We observed no fixation failures in any of the treated animals, however further work is required to better characterise to pull-out strength of these implants. In preliminary pilot studies performed by our research group, which examined traditional chondral fixation methods in both the condyle and trochlea ridge in goats, we observed fixation failure to some degree in over 50% of cases after four weeks, which necessitated the need for the development of the alternative fixation methods presented here.

No aberrant changes in the opposing surface of the joint were observed as a result of implanting the scaffold and the supporting fixation device. Based on our pilot study (Supplementary Fig. 1), we observed that the head of the pin was covered by tissue within 4 weeks, which alleviates concerns that the pin itself might damage the opposing articular surface. In addition, in order to prevent against damage to the opposing joint surface and the development of potential complications such as patellofemoral articulating (kissing) lesions, the PCL ring at the head of the pin, which maintains the scaffold in position, consists of just a single fibre (∼200 ​μm Ø) which is highly flexible*.*Together our results provide support for the use of such a scaffold fixation in preclinical studies, and potentially in clinical practice, to address the current failings of scaffold fixation techniques within synovial joints. More detailed histological assessment of the opposing joint structures (articular cartilage and meniscus) is however warranted to confirm that no damage is occurring within these tissues due to the presence of the fixation pin. To assess the impact of the fixation device on the overall mechanical properties of the implant, mechanical testing of the implants (scaffold ​+ ​fixation device) was also performed prior to surgery (Supplementary Fig. 4). While, as expected, some reinforcement of the construct was observed as a result of integrating the PCL fixation device into the scaffold, the mechanical properties observed (mean compressive modulus of 39.6 ​kPa) were still substantially less than native articular cartilage and minimal mechanical support would be provided by the construct upon implantation *in vivo.*

MFX remains the first line treatment choice for many orthopaedic surgeons due to its low price-point and short surgical time [9,10]. Due to the fact that the repair tissue observed is predominantly fibro-cartilaginous in nature, biomechanical deterioration of the repair is usually observed between 18 and 24 months post-surgery [69]. This leads to high rates of revision surgery after MFX, although the figures in the literature vary, in some patient cohorts up to 25% of patients required revision surgery on average 18 months after the initial procedure [70]. In this study we demonstrate high animal-to-animal variation in the levels of repair following MFX in a caprine model of cartilage defect repair, mimicking what is observed in humans. While some animals have good macroscopic and histological outcomes, the repair tissue of approximately half of the animals stained weakly/negligible for sGAG and type II collagen. However, when MFX was used in combination with the AC-ECM scaffold we observed an improvement in the consistency of repair. Due to the fact that we performed bi-lateral surgery, treating each animal with either MFX or the AC-ECM scaffold, we could chart differences in repair in the individual animal as a result of scaffold implantation. We observed that in animals with a poor innate healing capacity, characterised here by a score of 2.5 points or less in the gross morphology scoring following MFX, that scaffold implantation led to the most dramatic improvements in repair. Although the exact mechanism behind this improvement remains to be elucidated, if translated to the human condition, this suggests that implantation of AC-ECM derived scaffolds may lead to more consistent and reliable cartilage regeneration following MFX surgery.

We observed positive lubricin staining in the superficial zone of defects treated with AC-ECM scaffolds, and consistently with TGF-β3 loaded constructs, a result not observed across all animals in the MFX group. Lubricin (also known as superficial zone protein (SZP) or proteoglycan 4 (PGR4)) is a key protein in healthy articular cartilage as it is responsible for coating the cartilage surface and providing boundary lubrication, thereby preventing damage to the superficial zone and preserving the chondrocytes beneath [71]. The TGF-β signalling pathway has been implicated in several studies for its role in promoting lubricin expression [72,73], possibly explaining the increase in lubricin expression observed in the TGF-β3 loaded scaffolds. In addition to lubricin expression, we also examined collagen fibre alignment in the superficial zone of the repair tissue to ascertain if scaffold implantation promoted recapitulation of zonally defined articular cartilage. This hierarchical structure of AC is responsible for the load bearing, wear resistance and shock absorption properties of the tissue [74]. We were able to demonstrate that the average angle of fibre orientation in the superficial region of the repair tissue, as well as the level of fibre dispersion, was more akin to the native tissue in scaffold treated defects. While we observed an improvement in collagen fibre alignment as a result of implanting the AC-ECM scaffold, substantial differences between the treatment groups and native tissue could still be visualized. Unlike the native tissue, well aligned collagen fibres were not observed in the deeper regions of the tissue (data not shown), although we have previously observed that such structural remodelling of deep zones of cartilage repair tissue does not occur until later (approximately 1 year post scaffold implantation) in the repair process [29]. Longer-term studies are therefore required to determine if implantation of AC-ECM scaffolds will lead to further improvements in collagen fibre orientation and the development of a mechanically functional tissue. Improvements in scaffold design, such as the use of directional pore orientation, could potentially accelerate the development of a zonally defined repair tissue and reduce the levels of fibre dispersion observed.

In order to advance this technology to the clinic, a number of study limitations must be addressed. A limitation of this study is the lack of a direct comparison to a clinically available, type I collagen based scaffold product. It should also be noted that the experiments performed in this study used research grade materials and processes. We envisage that our future studies will use good manufacturing practice (GMP) grade porcine tissue, pepsin and PCL in order to conclusively demonstrate to regulatory bodies that this device can be manufactured to the highest standard without compromising efficacy. The PCL that we used to fabricate the fixation device has a high molecular weight (MW 50,000), is nonporous and as such the degradation of the shaft of the pin would be expected to be minimal over the 6 months of the *in vivo* study. PCL of high MW (60,000) has been shown to degrade slowly over 2–3 years *in vivo* [75]. PCL has a well-established track record in medical devices and elicits minimum inflammatory and immunological responses [[76], [77], [78]]. PCL is considered bioresorbable, a concept which reflects complete elimination of the initial foreign materials and bulk degradation by-products with little or no residual side effects [76]. While the available preclinical data on long term (beyond 12 months) degradation and integration of PCL implants in musculoskeletal tissue repair models is somewhat limited, they have been evaluated in a rabbit calvaria defect model at two years. In the study in question; calcium deposition indicated remodelling via osteoblast penetration into the scaffold structure and micro-CT analysis demonstrated that new bone had replaced the scaffold struts in some areas [77]. While the PCL in our fixation pin would degrade slowly over several years, we would expect full integration of the implant, remodelling to bone and eventual total bioresorbtion of the implant. However, before this occurs the presence of the fixation device could have potential inhibitory effects on subchondral bone healing and implant integration which should not be discounted, motivating longer-term follow-up studies to address this question. Exploring faster-degrading polymers such as Poly(lactic acid) (PLA) and poly(lactic-co-glycolic acid) (PLGA) for the fixation device may be warranted to alleviate this concern.

Recently, concerns with the microfracture procedure itself have centred on the detrimental effects on subchondral bone restoration from drilling/puncturing into the subchondral bone [79,80]. Translational models of bone marrow stimulation techniques have demonstrated the benefits of using awls, picks and wires of small-diameter over larger instruments [[81], [82], [83], [84]]. In this study we employed the use of two different k-wires to perforate the subchondral bone. For the central hole, into which the fixation device was inserted a 1.6 ​mm Ø k-wire was used and a 0.7 ​mm Ø k-wire was used for the other four microfracture holes. In a one-month pilot study, we did not observe any aberrant changes in the subchondral bone when μCT imaging was performed (Supplementary Fig. 1) as a result of this technique. However, it is of note that in the animals with the worst levels of repair; incomplete remodelling or fibrous tissue can be observed at the top of the subchondral bone region, this is particularly noticeable in the centre of the defects for the MFX only group (without fixation device). In order to improve efficacy of the device, a reduction in the k-wire diameter for all holes may be beneficial, however, this would also require a reduction in the diameter of the fixation pin shaft. In the future this should be possible with further developments of 3D printing technology or the use of alternative manufacturing techniques (e.g., injection molding) to produce a smaller diameter pin.

Another limitation of this study was that we were unable to undertake a detailed characterization of the mechanical properties of the repair tissue formed *in vivo*. In our previous *in vitro* work where AC-ECM scaffolds were seeded with infrapatellar fat pad derived stromal cells, the mechanical properties of the engineered tissues were still noticeable lower than native articular cartilage after long-term culture [28]. Future studies should therefore look to examine the depth-dependant mechanical properties of the repair tissue at different timepoints post-implantation to assess how it compares to normal articular cartilage [85].

While the addition of TGF-β3 to the scaffold improved the quality of tissue repair *in vivo,* it should be noted that the use of such supraphysiological doses of delivered growth factors may result in additional regulatory hurdles. Methods that can control the release kinetics of growth factors from the scaffold in a spatiotemporal manner may allow for a reduction in total TGF-β3 dose required [86]. Despite the relatively small pore size of the AC-ECM derived scaffolds (∼20 ​μm [28]), the high porosity (98%), allows cells to readily penetrate into the centre of the scaffolds. Previous work in our group has evaluated the release of TGF-β3 from cartilage derived ECM scaffolds over time [42,87]. It was found that the majority of TGF-β3 that was loaded into the construct was released over the first 8–10 days of culture, with a burst release (of ∼25% of the total released) occurring within the first day of culture. On average 75% of the TGF-β3 was found to have been released after 7 days in culture. While the addition of TGF-β3 to the AC-ECM scaffolds was associated with a significant improvement in type II collagen deposition in the defect site, significant differences were not detected in other parameters examined, raising the question as to whether the addition of a growth factor into such a regenerative product is justified based on the data presented in this study. The use of TGF-β3, or indeed any growth factor, will greatly increase the regulatory challenges associated with translating a new regenerative therapy into the clinic and therefore further long-term studies are required in order to justify the use the of TGF-β3 loaded scaffolds over non-loaded AC-ECM scaffolds. This might include, for example, an investigation of whether the delivery of TGF-β3 facilities the regeneration of zonally organised hyaline cartilage in long term (e.g. 12 month) *in vivo* studies. It will also be necessary to determine the mechanism of action associated with growth factor delivery. Based on our *in vitro* data in which we observed a 4-fold increase in cell migration in TGF-β3 loaded scaffolds, we hypothesized that the addition of TGF-β3 to the scaffolds would promote the chemotactic homing of regenerative cells from tissues such as the synovium or bone marrow [49,51] into the scaffold when implanted. However, we did not observe any noticeable increases in cellularity of the defect site with scaffolds loaded with TGF-β3, warranting more detailed investigations at earlier timepoints to more definitively understand the mechanism by which *in vivo* growth factor delivery is improving the quality of repair. It is also possible that the benefits of TGF-β3 delivery are also due, at least in part, to the direct chondrogenic differentiation of recruited cells in response to growth factor stimulation [40] as opposed to increased cell homing/chemotaxis.

## 5 Conclusions

Cartilage repair is often referred to as the “holy grail” of orthopaedics and sports medicine. There is a significant and growing need for new approaches to AC regeneration, as all current therapeutic options are sub-optimal and surgical revision rates are unacceptably high. We have developed a new regenerative implant for AC repair with the potential to improve the clinical treatment of focal cartilage defects. We have demonstrated that this scaffold can more consistently promote hyaline cartilage repair in a clinically relevant large animal model. Furthermore, the device is easy to use, requires a short surgical time and is compatible with microfracture, the current standard of care, thereby increasing the likelihood of short-term clinical translation.

## Funding

Funding for the project was received from 10.13039/501100001588Enterprise Ireland (CF/2014/4325), 10.13039/501100001602Science Foundation Ireland (12/IA/1554 and 12/RC/2278_P2) and by the 10.13039/501100000781European Research Council (ANCHOR – 779,909, StemRepair −258,463 and JointPrinting – 647,004). DCB has also received funding from the Orthoregeneration Network (ON) foundation and the 10.13039/100009283Orthopaedic Research Society (ORS) through a Kick-Starter award. PJDP was funded by the Irish Research Council (GOIPG/2015/3186). FEF recieved funding from the European Union’s Horizon 2020 research and innovation programme under the Marie Skłodowska- Curie Grant Agreement No. 839150. This research was co-funded by the European Regional Development Fund (10.13039/501100008530ERDF) under Ireland's European Structural and Investment Funds Programmes 2014–2020.

## Credit author statement

**David C. Browe**: Conceptualization, Methodology, Writing – original draft. Visualization, Investigation, Formal analysis; **Ross Burdis**: Methodology, Investigation, Visualization, Writing – review & editing, **Pedro J. Díaz-Payno**: Methodology, Investigation, Writing – review & editing, **Fiona E. Freeman**: Methodology, Investigation, Writing – review & editing, **Jessica M. Nulty**: Investigation, Writing – review & editing, **Conor T. Buckley**: Writing – review & editing, Supervision, **Pieter A.J. Brama**: Writing – review & editing, Methodology, Project administration, **Daniel J. Kelly**: Conceptualization, Writing – review & editing, Methodology, Supervision, Project administration, Funding acquisition,

## Author contributions

DCB was responsible for the design and fabrication of scaffolds, *in vitro* testing, *in vivo* testing, data acquisition, data interpretation, histological analysis and drafting the manuscript. RB designed and fabricated the fixation device, assisted with surgeries and assisted with histological scoring. PJD-P provided the polarized light microscopy images and analysis, assisted with surgeries, assisted with histological scoring and edited the manuscript. FEF assisted with surgeries, assisted with histological scoring and edited the manuscript. JMN assisted with surgeries, assisted with histological scoring and edited the manuscript. CTB conceived and assisted with design of experiments and edited the manuscript. PAJB was responsible for development of the large animal model, assisted with design of experiments, was the surgeon for the study and edited the manuscript. DJK conceived and assisted with design of experiments, oversaw the collection of results and data interpretation, acquired the funding for the study and finalized the paper.

## Data and materials availability

All data needed to evaluate the conclusions in the paper are present in the paper and/or the Supplementary Materials. The raw/processed data required to reproduce these findings cannot be shared at this time as the data also forms part of an ongoing study.

## Declaration of competing interest

The authors declare the following financial interests/personal relationships which may be considered as potential competing interests: David C Browe, Conor T Buckley, Pedro J.Díaz-Payno and Daniel J Kelly have patent #EP3180043B1 issued to Trinity College Dublin. David C Browe, Conor T Buckley and Daniel J Kelly are co-founders and shareholders in Altach Biomedical Ltd. Altach did not sponsor this research. Research undertaken in Daniel Kelly’s laboratory at Trinity College Dublin is part-funded by Johnson & Johnson services (J&J). J&J did not sponsor this study.

## References

[1] Brittberg M. (2018). Clinical articular cartilage repair—an up to date review. Annals of Joint.

[2] Smith G.D., Knutsen G., Richardson J.B. (2005). A clinical review of cartilage repair techniques. J Bone Joint Surg Br.

[3] Lories R.J., Luyten F.P. (2011). The bone-cartilage unit in osteoarthritis. Nat. Rev. Rheumatol..

[4] Mankin H.J. (1974). The reaction of articular cartilage to injury and osteoarthritis (second of two parts). N. Engl. J. Med..

[5] Hunter D.J., Bierma-Zeinstra S. (2019). Osteoarthritis. Lancet.

[6] Salmon J.H., Rat A.C., Sellam J., Michel M., Eschard J.P., Guillemin F., Jolly D., Fautrel B. (2016). Economic impact of lower-limb osteoarthritis worldwide: a systematic review of cost-of-illness studies. Osteoarthritis Cartilage.

[7] Woolf A.D., Pfleger B. (2003). Burden of major musculoskeletal conditions. Bull. World Health Organ..

[8] Spahn G., Hofmann G.O. (2014). [Focal cartilage defects within the medial knee compartment. predictors for osteoarthritis progression]. Z. für Orthop. Unfallchirurgie.

[9] Makris E.A., Gomoll A.H., Malizos K.N., Hu J.C., Athanasiou K.A. (2015). Repair and tissue engineering techniques for articular cartilage. Nat. Rev. Rheumatol..

[10] Steadman J.R., Rodkey W.G., Briggs K.K., Rodrigo J.J. (1999). [The microfracture technic in the management of complete cartilage defects in the knee joint]. Orthopä.

[11] Pestka J.M., Bode G., Salzmann G., Sudkamp N.P., Niemeyer P. (2012). Clinical outcome of autologous chondrocyte implantation for failed microfracture treatment of full-thickness cartilage defects of the knee joint. Am. J. Sports Med..

[12] Demange M.K., Minas T., Gomoll A.H. (2014).

[13] Everhart J.S., Jiang E.X., Poland S.G., Du A., Flanigan D.C. (2019). Failures, reoperations, and improvement in knee symptoms following matrix-assisted autologous chondrocyte transplantation. A Meta-Analysis of Prospective Comparative Trials, Cartilage.

[14] Na Y., Shi Y., Liu W., Jia Y., Kong L., Zhang T., Han C., Ren Y. (2019). Is implantation of autologous chondrocytes superior to microfracture for articular-cartilage defects of the knee? A systematic review of 5-year follow-up data. Int. J. Surg..

[15] Behrens P., Bitter T., Kurz B., Russlies M. (2006). Matrix-associated autologous chondrocyte transplantation/implantation (MACT/MACI)--5-year follow-up. Knee.

[16] Cherubino P., Grassi F., Bulgheroni P., M.J.J.o.O.S. Ronga (2003). Autologous chondrocyte implantation using a bilayer collagen membrane. a preliminary report.

[17] Driscoll D., Farnia S., Kefalas P., Maziarz R.T. (2017). Concise review: the high cost of high tech medicine: planning ahead for market access. Stem Cells Transl Med.

[18] Familiari F., Cinque M.E., Chahla J., Godin J.A., Olesen M.L., Moatshe G., LaPrade R.F. (2018). Clinical outcomes and failure rates of osteochondral allograft transplantation in the knee: a systematic review. Am. J. Sports Med..

[19] Aae T.F., Randsborg P.-H., Lurås H., Årøen A., Lian Ø.B. (2018). Microfracture is more cost-effective than autologous chondrocyte implantation: a review of level 1 and level 2 studies with 5 year follow-up. Knee Surg. Sports Traumatol. Arthrosc..

[20] Kraeutler M.J., Belk J.W., Purcell J.M., McCarty E.C. (2018). Microfracture versus autologous chondrocyte implantation for articular cartilage lesions in the knee: a systematic review of 5-year outcomes. Am. J. Sports Med..

[21] Martín A.R., Patel J.M., Zlotnick H.M., Carey J.L., Mauck R.L. (2019). Emerging therapies for cartilage regeneration in currently excluded ‘red knee’populations. NPJ Regenerative medicine.

[22] Zhang Y., He Y., Bharadwaj S., Hammam N., Carnagey K., Myers R., Atala A., Van Dyke M. (2009). Tissue-specific extracellular matrix coatings for the promotion of cell proliferation and maintenance of cell phenotype. Biomaterials.

[23] Badylak S.F. (2007). The extracellular matrix as a biologic scaffold material. Biomaterials.

[24] Huang B.J., Hu J.C., Athanasiou K.A. (2016). Cell-based tissue engineering strategies used in the clinical repair of articular cartilage. Biomaterials.

[25] Tamaddon M., Burrows M., Ferreira S.A., Dazzi F., Apperley J.F., Bradshaw A., Brand D.D., Czernuszka J., Gentleman E. (2017). Monomeric, porous type II collagen scaffolds promote chondrogenic differentiation of human bone marrow mesenchymal stem cells in vitro. Sci. Rep..

[26] Yang K., Sun J., Wei D., Yuan L., Yang J., Guo L., Fan H., Zhang X. (2017). Photo-crosslinked mono-component type II collagen hydrogel as a matrix to induce chondrogenic differentiation of bone marrow mesenchymal stem cells. J. Mater. Chem. B.

[27] Sutherland A.J., Converse G.L., Hopkins R.A., Detamore M.S. (2015). The bioactivity of cartilage extracellular matrix in articular cartilage regeneration. Adv Healthc Mater.

[28] Browe D.C., Mahon O.R., Diaz-Payno P.J., Cassidy N., Dudurych I., Dunne A., Buckley C.T., Kelly D.J. (2019). Glyoxal cross-linking of solubilized extracellular matrix to produce highly porous, elastic, and chondro-permissive scaffolds for orthopedic tissue engineering. J. Biomed. Mater. Res..

[29] Cunniffe G.M., Diaz-Payno P.J., Sheehy E.J., Critchley S.E., Almeida H.V., Pitacco P., Carroll S.F., Mahon O.R., Dunne A., Levingstone T.J., Moran C.J., Brady R.T., O'Brien F.J., Brama P.A.J., Kelly D.J. (2019). Tissue-specific extracellular matrix scaffolds for the regeneration of spatially complex musculoskeletal tissues. Biomaterials.

[30] Yang Q., Peng J., Guo Q., Huang J., Zhang L., Yao J., Yang F., Wang S., Xu W., Wang A., Lu S. (2008). A cartilage ECM-derived 3-D porous acellular matrix scaffold for in vivo cartilage tissue engineering with PKH26-labeled chondrogenic bone marrow-derived mesenchymal stem cells. Biomaterials.

[31] Zhang Y., Liu S., Guo W., Wang M., Hao C., Gao S., Zhang X., Li X., Chen M., Jing X., Wang Z., Peng J., Lu S., Guo Q. (2018). Human umbilical cord Wharton's jelly mesenchymal stem cells combined with an acellular cartilage extracellular matrix scaffold improve cartilage repair compared with microfracture in a caprine model. Osteoarthritis Cartilage.

[32] Díaz-Payno P.J., Browe D.C., Cunniffe G.M., Kelly D.J.J.B., Communications B.R. (2020).

[33] Hjelle K., Solheim E., Strand T., Muri R., Brittberg M. (2002). Articular cartilage defects in 1,000 knee arthroscopies. Arthroscopy.

[34] Bekkers J.E., Tsuchida A.I., Malda J., Creemers L.B., Castelein R.J., Saris D.B., Dhert W.J. (2010). Quality of scaffold fixation in a human cadaver knee model. Osteoarthritis Cartilage.

[35] Drobnic M., Radosavljevic D., Ravnik D., Pavlovcic V., Hribernik M. (2006). Comparison of four techniques for the fixation of a collagen scaffold in the human cadaveric knee. Osteoarthritis Cartilage.

[36] Patel J.M., Sennett M.L., Martin A.R., Saleh K.S., Eby M.R., Ashley B.S., Miller L.M., Dodge G.R., Burdick J.A., Carey J.L. (2021). Resorbable pins to enhance scaffold retention in a porcine chondral defect model. Cartilage.

[37] Mancini I.A.D., Vindas Bolaños R.A., Brommer H., Castilho M., Ribeiro A., van Loon J.P.A.M., Mensinga A., van Rijen M.H.P., Malda J., van Weeren R. (2017). Fixation of hydrogel constructs for cartilage repair in the equine model: a challenging issue, tissue engineering. Part C, Methods.

[38] Deyl Z., Miksik I., Eckhardt A. (2003). Preparative procedures and purity assessment of collagen proteins. J. Chromatogr., B: Anal. Technol. Biomed. Life Sci..

[39] Freeman F.E., Browe D.C., Nulty J., Von Euw S., Grayson W.L., Kelly D.J. (2019). Biofabrication of multiscale bone extracellular matrix scaffolds for bone tissue engineering. Eur. Cell. Mater..

[40] Johnstone B., Hering T.M., Caplan A.I., Goldberg V.M., Yoo J.U. (1998). In vitro chondrogenesis of bone marrow-derived mesenchymal progenitor cells. Exp. Cell Res..

[41] Browe D.C., Coleman C.M., Barry F.P., Elliman S.J. (2019). Hypoxia activates the PTHrP–MEF2C pathway to attenuate hypertrophy in mesenchymal stem cell derived cartilage. Sci. Rep..

[42] Almeida H.V., Mulhall K.J., O'Brien F.J., Kelly D.J. (2017). Stem cells display a donor dependent response to escalating levels of growth factor release from extracellular matrix-derived scaffolds. J Tissue Eng Regen Med.

[43] Sheehy E.J., Mesallati T., Vinardell T., Kelly D.J. (2015). Engineering cartilage or endochondral bone: a comparison of different naturally derived hydrogels. Acta Biomater..

[44] Mainil-Varlet P., Van Damme B., Nesic D., Knutsen G., Kandel R., Roberts S. (2010). A new histology scoring system for the assessment of the quality of human cartilage repair: ICRS II. Am. J. Sports Med..

[45] Reznikov N., Almany-Magal R., Shahar R., Weiner S. (2013). Three-dimensional imaging of collagen fibril organization in rat circumferential lamellar bone using a dual beam electron microscope reveals ordered and disordered sub-lamellar structures. Bone.

[46] Levingstone T.J., Ramesh A., Brady R.T., Brama P.A.J., Kearney C., Gleeson J.P., O'Brien F.J. (2016). Cell-free multi-layered collagen-based scaffolds demonstrate layer specific regeneration of functional osteochondral tissue in caprine joints. Biomaterials.

[47] Getgood A.M., Kew S.J., Brooks R., Aberman H., Simon T., Lynn A.K., N.J.T.K. Rushton (2012). Evaluation of early-stage osteochondral defect repair using a biphasic scaffold based on a collagen. glycosaminoglycan biopolymer in a caprine model.

[48] Tang Y., Wu X., Lei W., Pang L., Wan C., Shi Z., Zhao L., Nagy T.R., Peng X., Hu J., Feng X., Van Hul W., Wan M., Cao X. (2009). TGF-beta1-induced migration of bone mesenchymal stem cells couples bone resorption with formation. Nat. Med..

[49] Mendelson A., Frank E., Allred C., Jones E., Chen M., Zhao W., Mao J.J. (2011). Chondrogenesis by chemotactic homing of synovium, bone marrow, and adipose stem cells in vitro. Faseb. J..

[50] Deng M., Mei T., Hou T., Luo K., Luo F., Yang A., Yu B., Pang H., Dong S., Xu J. (2017). TGFbeta3 recruits endogenous mesenchymal stem cells to initiate bone regeneration. Stem Cell Res. Ther..

[51] Lee C.H., Cook J.L., Mendelson A., Moioli E.K., Yao H., Mao J.J. (2010). Regeneration of the articular surface of the rabbit synovial joint by cell homing: a proof of concept study. Lancet.

[52] Crawford D.C., DeBerardino T.M., Williams R.J. (2012). NeoCart, an autologous cartilage tissue implant, compared with microfracture for treatment of distal femoral cartilage lesions: an FDA phase-II prospective, randomized clinical trial after two years. J Bone Joint Surg Am.

[53] Zak L., Albrecht C., Wondrasch B., Widhalm H., Vekszler G., Trattnig S., Marlovits S., Aldrian S. (2014). Results 2 Years after matrix-associated autologous chondrocyte transplantation using the Novocart 3D scaffold: an analysis of clinical and radiological data. Am. J. Sports Med..

[54] D'Ambrosi R., Valli F., De Luca P., Ursino N., Usuelli F.G. (2019). MaioRegen osteochondral substitute for the treatment of knee defects: a systematic review of the literature. J. Clin. Med..

[55] Schneider U., Rackwitz L., Andereya S., Siebenlist S., Fensky F., Reichert J., Loer I., Barthel T., Rudert M., Noth U. (2011). A prospective multicenter study on the outcome of type I collagen hydrogel-based autologous chondrocyte implantation (CaReS) for the repair of articular cartilage defects in the knee. Am. J. Sports Med..

[56] Luo Y., Sinkeviciute D., He Y., Karsdal M., Henrotin Y., Mobasheri A., Onnerfjord P., Bay-Jensen A. (2017). The minor collagens in articular cartilage. Protein Cell.

[57] Mahon O.R., Browe D.C., Diaz-Payno P.J., Pitacco P., Cunningham K.T., Mills K.H.G., Dunne A., Kelly D.J. (2021). Extracellular matrix scaffolds derived from different musculoskeletal tissues drive distinct macrophage phenotypes and direct tissue-specific cellular differentiation. Journal of Immunology and Regenerative Medicine.

[58] Yoon D.S., Lee K.M., Kim S.H., Kim S.H., Jung Y., Kim S.H., Park K.H., Choi Y., Ryu H.A., Choi W.J., Lee J.W. (2016). Synergistic action of IL-8 and bone marrow concentrate on cartilage regeneration through upregulation of chondrogenic transcription factors. Tissue Eng..

[59] Kim J.H., Lee M.C., Seong S.C., Park K.H., Lee S. (2010). Enhanced proliferation and chondrogenic differentiation of human synovium-derived stem cells expanded with basic fibroblast growth factor. Tissue Eng..

[60] Gawlitta D., Benders K.E., Visser J., van der Sar A.S., Kempen D.H., Theyse L.F., Malda J., Dhert W.J. (2015). Decellularized cartilage-derived matrix as substrate for endochondral bone regeneration. Tissue Eng..

[61] Daly A.C., Cunniffe G.M., Sathy B.N., Jeon O., Alsberg E., Kelly D.J. (2016). 3D bioprinting of developmentally inspired templates for whole bone organ engineering. Adv Healthc Mater.

[62] Freeman F.E., Brennan M.Á., Browe D.C., Renaud A., De Lima J., Kelly D.J., McNamara L.M., Layrolle P. (2020). A developmental engineering-based approach to bone repair: endochondral priming enhances vascularization and new bone formation in a critical size defect. Front. Bioeng. Biotechnol..

[63] Cunniffe G.M., Diaz-Payno P.J., Ramey J.S., Mahon O.R., Dunne A., Thompson E.M., O'Brien F.J., Kelly D.J. (2017). Growth plate extracellular matrix-derived scaffolds for large bone defect healing. Eur. Cell. Mater..

[64] Browe D.C., Díaz-Payno P.J., Freeman F.E., Schipani R., Burdis R., Ahern D.P., Nulty J.M., Guler S., Randall L.D., Buckley C.T., Brama P.A.J., Kelly D.J. (2022). Bilayered extracellular matrix derived scaffolds with anisotropic pore architecture guide tissue organization during osteochondral defect repair. Acta Biomater..

[65] Hunziker E.B., Stahli A. (2008). Surgical suturing of articular cartilage induces osteoarthritis-like changes. Osteoarthritis Cartilage.

[66] Knecht S., Erggelet C., Endres M., Sittinger M., Kaps C., Stussi E. (2007). Mechanical testing of fixation techniques for scaffold-based tissue-engineered grafts. J. Biomed. Mater. Res. B Appl. Biomater..

[67] Brittberg M., Sjögren-Jansson E., Lindahl A., Peterson L.J.B. (1997). Influence of fibrin sealant (Tisseel®). on osteochondral defect repair in the rabbit knee.

[68] Friedman J.M., Sennett M.L., Bonadio M.B., Orji K.O., Neuwirth A.L., Keah N., Carey J.L., Moutos F.T., Estes B.T., Guilak F., Madry H., Mauck R.L., Dodge G.R. (2018). Comparison of fixation techniques of 3D-woven poly(-caprolactone) scaffolds for cartilage repair in a weightbearing porcine large animal model. Cartilage.

[69] Kreuz P.C., Steinwachs M.R., Erggelet C., Krause S.J., Konrad G., Uhl M., Sudkamp N. (2006). Results after microfracture of full-thickness chondral defects in different compartments in the knee. Osteoarthritis Cartilage.

[70] Salzmann G.M., Sah B., Sudkamp N.P., Niemeyer P. (2013). Reoperative characteristics after microfracture of knee cartilage lesions in 454 patients. Knee Surg. Sports Traumatol. Arthrosc..

[71] Jay G.D., Waller K.A. (2014). The biology of lubricin: near frictionless joint motion. Matrix Biol..

[72] Schmidt T.A., Gastelum N.S., Han E.H., Nugent-Derfus G.E., Schumacher B.L., Sah R.L. (2008). Differential regulation of proteoglycan 4 metabolism in cartilage by IL-1alpha, IGF-I, and TGF-beta1. Osteoarthritis Cartilage.

[73] Lee S.Y., Niikura T., Reddi A.H. (2008). Superficial zone protein (lubricin) in the different tissue compartments of the knee joint: modulation by transforming growth factor beta 1 and interleukin-1 beta. Tissue Eng..

[74] Lu X.L., Mow V.C. (2008). Biomechanics of articular cartilage and determination of material properties. Med. Sci. Sports Exerc..

[75] Sun H., Mei L., Song C., Cui X., Wang P. (2006). The in vivo degradation, absorption and excretion of PCL-based implant. Biomaterials.

[76] Woodruff M.A., Hutmacher D.W. (2010). The return of a forgotten polymer—polycaprolactone in the 21st century. Prog. Polym. Sci..

[77] Lam C.X., Hutmacher D.W., Schantz J.T., Woodruff M.A., Teoh S.H. (2009). Evaluation of polycaprolactone scaffold degradation for 6 months in vitro and in vivo. J. Biomed. Mater. Res. Part A: An Official Journal of The Society for Biomaterials, The Japanese Society for Biomaterials, and The Australian Society for Biomaterials and the Korean Society for Biomaterials.

[78] Youssef A., Hollister S.J., Dalton P.D. (2017). Additive manufacturing of polymer melts for implantable medical devices and scaffolds. Biofabrication.

[79] Madry H., Orth P., Cucchiarini M. (2016). Role of the subchondral bone in articular cartilage degeneration and repair. JAAOS - Journal of the American Academy of Orthopaedic Surgeons.

[80] Chu C.R., Fortier L.A., Williams A., Payne K.A., McCarrel T.M., Bowers M.E., Jaramillo D. (2018). Minimally manipulated bone marrow concentrate compared with microfracture treatment of full-thickness chondral defects: a one-year study in an equine model. J Bone Joint Surg Am.

[81] Orth P., Duffner J., Zurakowski D., Cucchiarini M., Madry H. (2016). Small-diameter awls improve articular cartilage repair after microfracture treatment in a translational animal model. Am. J. Sports Med..

[82] Eldracher M., Orth P., Cucchiarini M., Pape D., Madry H. (2014). Small subchondral drill holes improve marrow stimulation of articular cartilage defects. Am. J. Sports Med..

[83] Zlotnick H., Locke R., Stoeckl B., Patel J., Gupta S., Browne K., Koh J., Carey J., Mauck R. (2021). Marked differences in local bone remodelling in response to different marrow stimulation techniques in a large animal. Eur. Cell. Mater..

[84] Zedde P., Cudoni S., Giachetti G., Manunta M.L., Masala G., Brunetti A., Manunta A.F. (2016). Subchondral bone remodeling: comparing nanofracture with microfracture. An ovine in vivo study. Joints.

[85] Gannon A.R., Nagel T., Bell A.P., Avery N.C., Kelly D.J. (2015). Postnatal changes to the mechanical properties of articular cartilage are driven by the evolution of its collagen network. Eur. Cell. Mater..

[86] Freeman F.E., Pitacco P., van Dommelen L.H., Nulty J., Browe D.C., Shin J.-Y., Alsberg E., Kelly D.J. (2020). 3D bioprinting spatiotemporally defined patterns of growth factors to tightly control tissue regeneration. Sci. Adv..

[87] Almeida H.V., Liu Y., Cunniffe G.M., Mulhall K.J., Matsiko A., Buckley C.T., O'Brien F.J., Kelly D.J. (2014). Controlled release of transforming growth factor-beta3 from cartilage-extra-cellular-matrix-derived scaffolds to promote chondrogenesis of human-joint-tissue-derived stem cells. Acta Biomater..

