# Supplementary material for: Promoting endogenous articular cartilage regeneration using extracellular matrix scaffolds
Source: Mater Today Bio. 2022 Jul 5;16:100343. doi: 10.1016/j.mtbio.2022.100343 (PMC9294195; doi:10.1016/j.mtbio.2022.100343)
Supplement: Multimedia component 1 [file mmc1.docx]

**Supplementary Data**


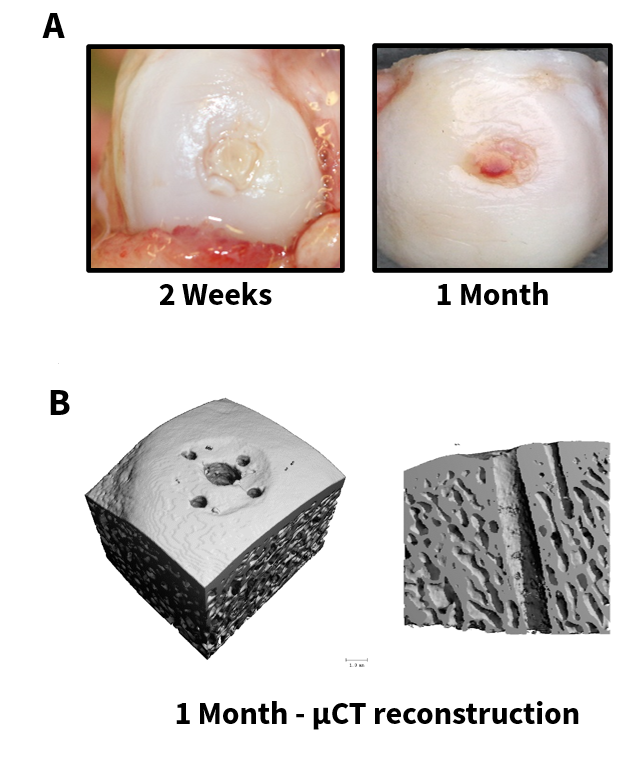


**Supplementary Fig 1**: Macroscopic images from pilot animal studies demonstrating the maintenance of the fixation device and scaffold after both 2 weeks and 1 month *in vivo* **(A)**. µCT reconstruction of defect site and microfracture drilling into subchondral bone after 1 month in a pilot animal. Scale bar = 1mm **(B).**


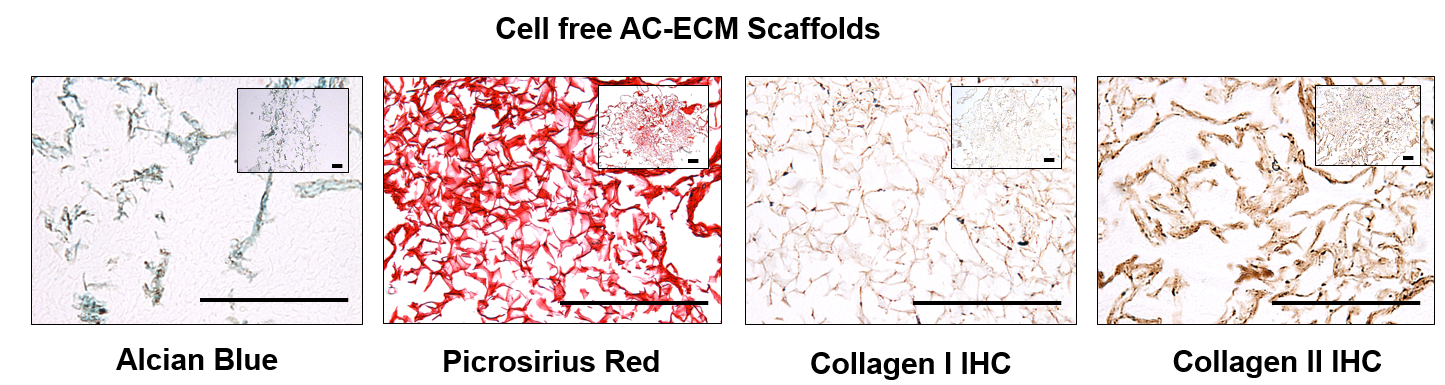


**Supplementary Fig 2**: Histology of cell free AC-ECM scaffolds. GAG deposition (Alcian Blue), collagen deposition (Picrosirius Red) and type I/II collagen presence was evaluated by histological / immunohistochemical staining. Scale bars = 200μm.


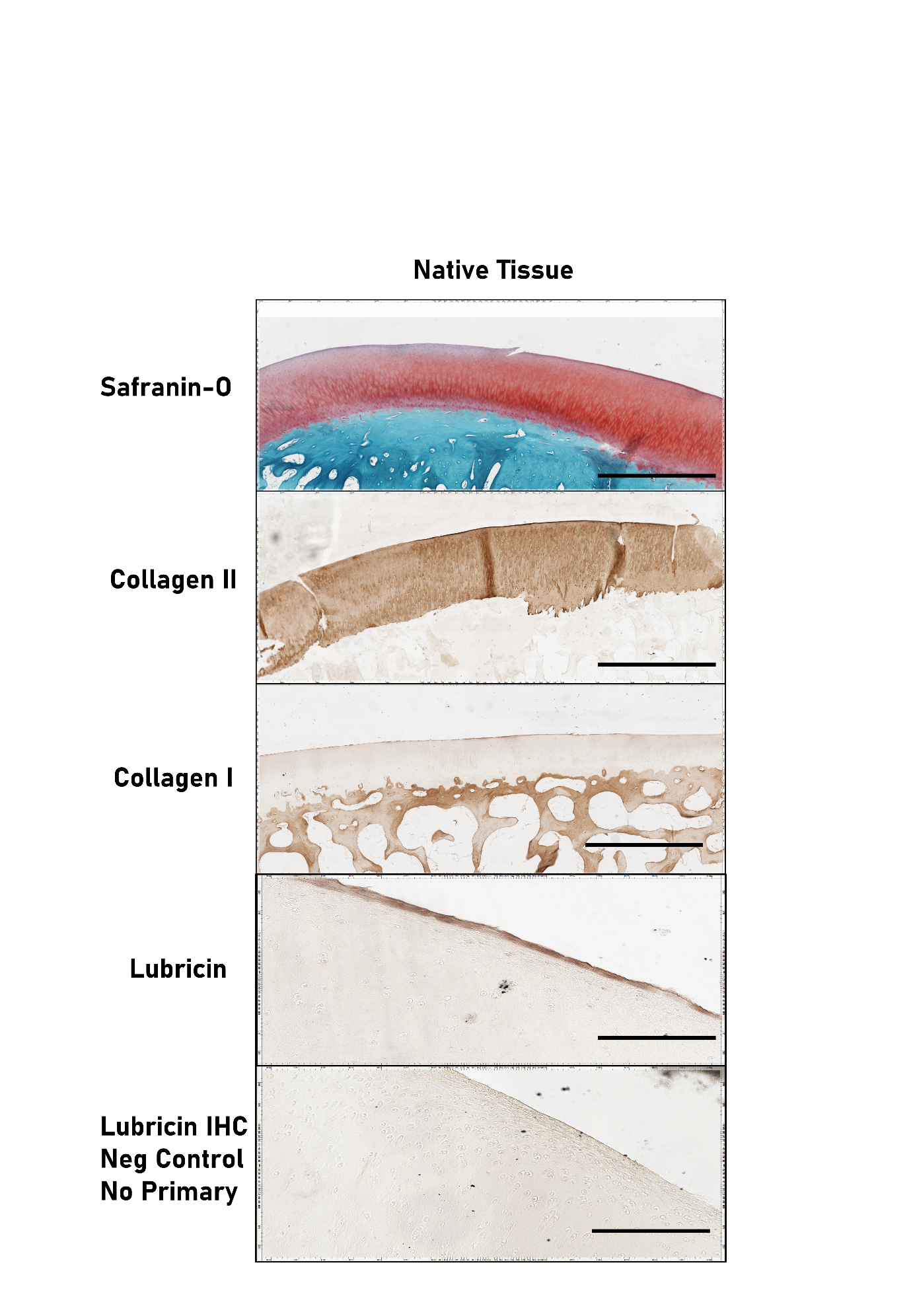


**Supplementary Fig 3**: Histological and immunohistochemical staining of native (untreated) goat condyles. Scale bar =2mm in Saf-O, Col I and Col II and 200μm for Lubricin IHC


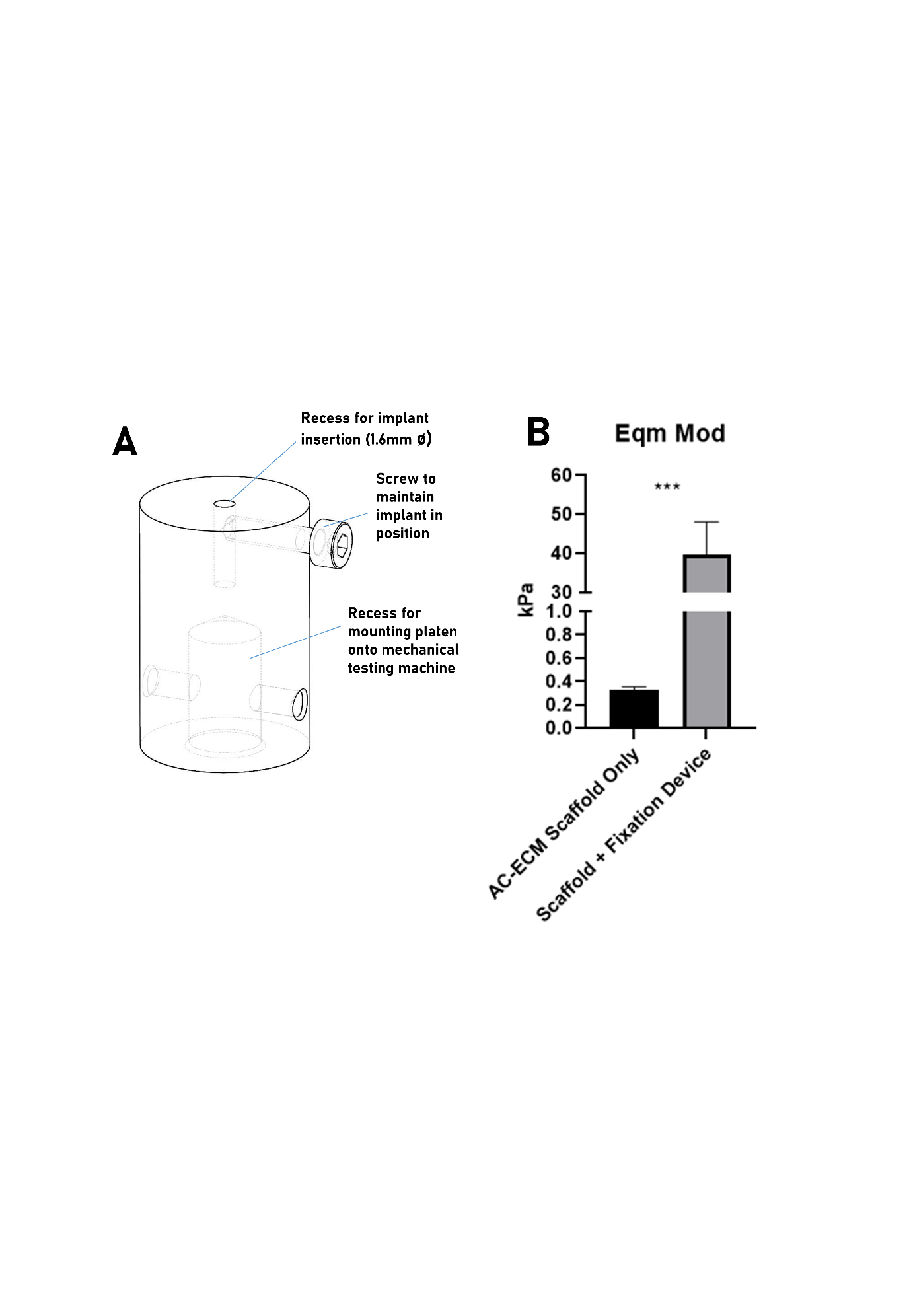


**Supplementary Fig 4**: Mechanical testing of implants prior to in vivo study. A custom designed, 3D printed platen was fabricated so that the shaft of the implant could be recessed within the platen while being maintained in position (**A**). To obtain the AC-ECM scaffold and scaffold + fixation device equilibrium modulus, stress relaxation tests were performed whereby 10% strain was applied to the samples (**B**).

|  | Tissue Morphology | Matrix Staining | Cell Morphology | Surface Architecture | Basal Intergradation | Tidemark | Overall assessment |
| --- | --- | --- | --- | --- | --- | --- | --- |
| Microfracture | 48.5 ±32.3 | 54.5 ±32.1 | 50.2 ±26.7 | 34.4 ± 31.1 | 53.1 ± 40.4 | 18.75 ± 40.1 | 34.5 ± 31.5 |
| AC-ECM Scaffold | 48.3 ± 28.4 | 64.38 ±20.2 | 75.8± 13.9 | 55.08 ± 23.06 | 53.23± 34.92 | 28.125 ± 35.94 | 50.79 ± 21.61 |
| Scaffold +TGF-β3 | 50.64 ± 25.08 | 45.53 ± 29.29 | 63.21 ± 27.00 | 49.46 ± 27.79 | 32.57 ± 27.11 | 17.32 ± 34.07 | 50.78 ± 28.35 |

**Supplementary Table 1**: Blinded histological scoring of the preclinical study using an adapted scoring system from the International Cartilage Repair Society (ICRS) II parameters.

| **Gene** | **Sequence 5′-3′** | **Accession Number** |
| --- | --- | --- |
| *GAPDH* | Fwd CTTTTGCGTCGCCAG Rev TTGATGGCAACAATATCCAC | NM_002046 |
| *Col1a1* | Fwd GATTCCCTGGACCTAAAGGTGC Rev AGCCTCTCCATCTTTGCCAGCA | NM_000088 |
| *Sox9* | Fwd CTCTGGAGACTTCTGAACG Rev AGATGTGCGTCTGCTC | NM_000346 |
| *Col2a1* | Fwd GAAGAGTGGAGACTACTGG Rev CAGATGTGTTTCTTCTCCTTG | NM_033150 |

**Supplementary Table 2**: Primer sequences used for Q-PCR
